# Excited-State (Anti)Aromaticity Explains Why Azulene Disobeys Kasha’s Rule

**DOI:** 10.1021/jacs.3c07625

**Published:** 2023-09-13

**Authors:** David Dunlop, Lucie Ludvíková, Ambar Banerjee, Henrik Ottosson, Tomáš Slanina

**Affiliations:** † Institute of Organic Chemistry and Biochemistry of the Czech Academy of Sciences, Flemingovo náměstí 542/2, Prague 6 160 00, Czech Republic; ‡ Department of Inorganic Chemistry, Faculty of Science, Charles University in Prague, Hlavova 2030, Prague 2 128 40, Czech Republic; § Division of X-ray Photon Science, Department of Physics and Astronomy—Ångström Laboratory, Uppsala University, Box 523, Uppsala 751 20, Sweden; ∥ Department of Chemistry—Ångström Laboratory, Uppsala University, Box 516, Uppsala 751 20, Sweden

## Abstract

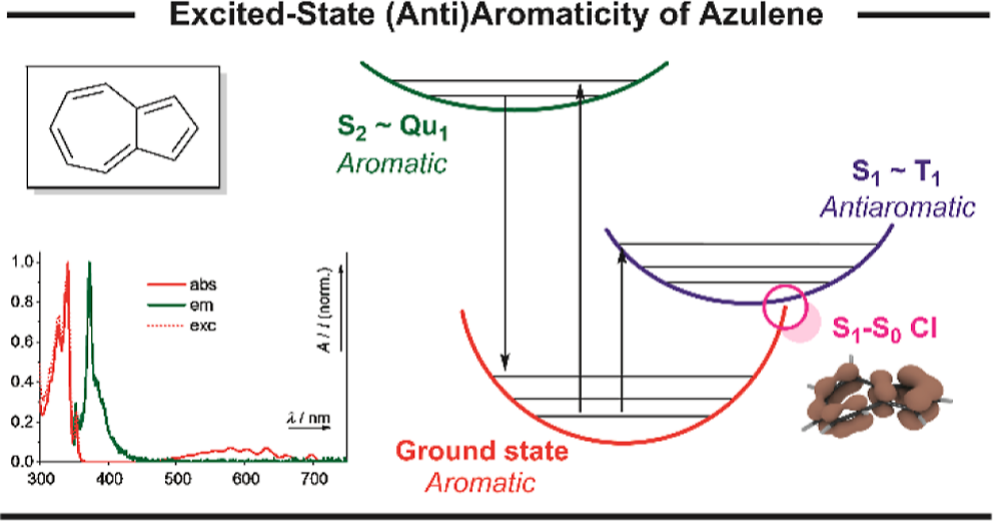

Fluorescence exclusively occurs from the lowest excited state of a given multiplicity according to Kasha’s rule. However, this rule is not obeyed by a handful of anti-Kasha fluorophores whose underlying mechanism is still understood merely on a phenomenological basis. This lack of understanding prevents the rational design and property-tuning of anti-Kasha fluorophores. Here, we propose a model explaining the photophysical properties of an archetypal anti-Kasha fluorophore, azulene, based on its ground- and excited-state (anti)aromaticity. We derived our model from a detailed analysis of the electronic structure of the ground singlet, first excited triplet, and quintet states and of the first and second excited singlet states using the perturbational molecular orbital theory and quantum-chemical aromaticity indices. Our model reveals that the anti-Kasha properties of azulene and its derivatives result from (i) the contrasting (anti)aromaticity of its first and second singlet excited states (S_1_ and S_2_, respectively) and (ii) an easily accessible antiaromaticity relief pathway of the S_1_ state. This explanation of the fundamental cause of anti-Kasha behavior may pave the way for new classes of anti-Kasha fluorophores and materials with long-lived, high-energy excited states.

## 1 Introduction

In 1959, Michael Kasha postulated that, in single molecules, “the emitting level of a given multiplicity is the lowest excited level of that multiplicity”.^1^ Since then, however, Kasha’s rule has been broken by a number of molecules known as anti-Kasha (also non-Kasha) fluorophores.^2–4^ Among them, one fluorophore stands out for exclusively emitting from the second singlet excited state (S_2_) as the archetype of anti-Kasha fluorophores, azulene.^5^

**Figure 1:**
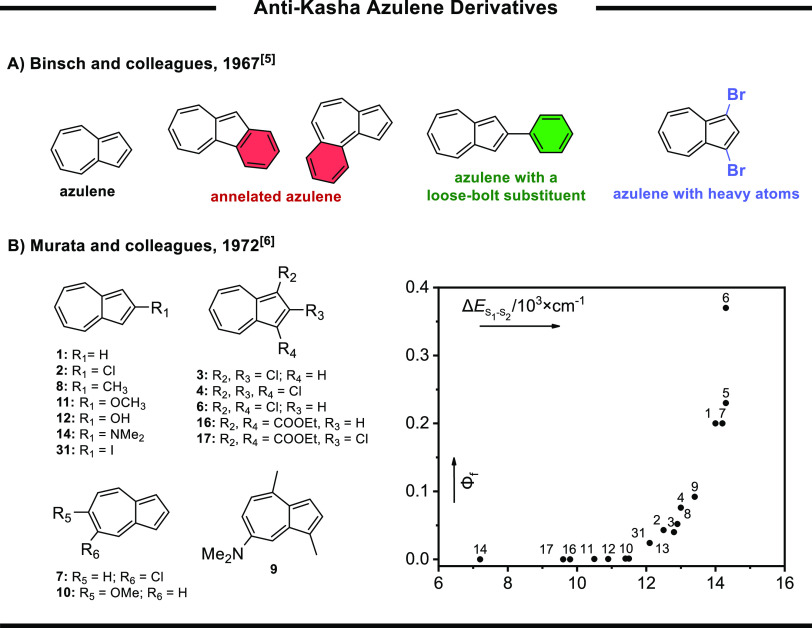
Examples of anti-Kasha azulene derivatives: (A) substituent effects explored by Binsch et al.,^6^ (B) derivatives (numbered as in the original publication) explored by Murata et al. (left) and a plot of fluorescence quantum yields as a function of S_1_–S_2_ gap (right).^7^

Azulene’s anti-Kasha behavior is particularly robust under structural perturbations. Binsch et al. tried to quell the anti-Kasha properties of azulene through multiple synthetic strategies, including annellation, symmetry lowering, substitution by heavy atoms, and addition of a “loose bolt” substituent (Figure 1A), albeit to no avail.^6^ All approaches failed to induce the first singlet excited state (S_1_) emission of the azulene derivatives.

Based on Longuet-Higgins and Beer’s hypothesis according to which the anomalous emission of azulene results from its large S_2_–S_1_ gap (∼14,000 cm^–1^),^5^ Murata et al. followed a more systematic approach to reduce the S_2_–S_1_ gap by extensive substitution of the azulene scaffold (Figure 1B).^7^ They were able to increase the rate of S_2_–S_1_ internal conversion, thereby reducing the quantum yield of the S_2_ emission. Yet, even in later studies, despite the increased IC rate, no S_1_ emission was observed in any azulene derivative at room temperature.^8–15^

The anomalous photophysical properties of azulene and its derivatives prompted further research efforts to uncover their underlying mechanism, leading to the following, now well-established explanations: (i) the large S_2_–S_1_ gap of azulene results in a low rate of IC from S_2_, which in turn leads to a high yield of S_2_ emission,^5,7,10^ and (ii) the S_1_ of azulene rapidly decays by a S_1_–S_0_ conical intersection,^18^ located near the S_1_ minimum energy geometry, thereby accounting for the absence of S_1_ emission. Notwithstanding these efforts, no structure–property relationship was provided to explain the anti-Kasha behavior of azulene. In fact, the description of azulene’s anti-Kasha behavior has long remained insufficient to guide any attempt at rational molecular design and anti-Kasha property tuning, a shortcoming that we shall overcome herein.

Azulene is a 10π-aromatic fused bicyclic hydrocarbon with no substituents or heteroatoms; therefore, its photophysical phenomena must be a manifestation of its π-electron configuration. Cyclic, π-conjugated molecules can be described as aromatic or antiaromatic in their ground and excited states.^19–24^ Among other characteristics, these descriptors indicate whether their π-electron configuration in a given electronic state is stabilizing or destabilizing, respectively. Accordingly, we hypothesized that the anti-Kasha behavior of azulene could be related to the (anti)aromatic character of S_1_ and S_2_. Such a relationship between excited-state (anti)aromaticity and anti-Kasha behavior may explain how the electronic structure of azulene leads to its anti-Kasha behavior, thus providing key mechanistic insights into anti-Kasha fluorophores.

## 2 Results and Discussion

To understand how the electronic structure of azulene leads to its anti-Kasha behavior, we analyzed its ground- and excited-state (anti)aromaticity in increasing order of complexity. The ground singlet state (S_0_) and first-excited triplet state (T_1_) of small conjugated cyclic hydrocarbons typically show contrasting aromaticity/antiaromaticity, as per Hückel’s and Baird’s rules.^20^ Moreover, the aromaticity of the first excited quintet state (Qu_1_) of azulene has been previously reported.^25,26^ Hence, we computationally investigated the S_0_, T_1_, and Qu_1_ to model the (anti)aromatic character of azulene in the lowest states of each multiplicity. Subsequently, we compared their electronic structures and various aromaticity indices (Section S5) with those of S_1_ and S_2_, which account for the anti-Kasha behavior of azulene. This approach enabled us to establish the relationship between the anti-Kasha behavior of azulene and its excited-state (anti)aromaticity.

**Figure 2:**
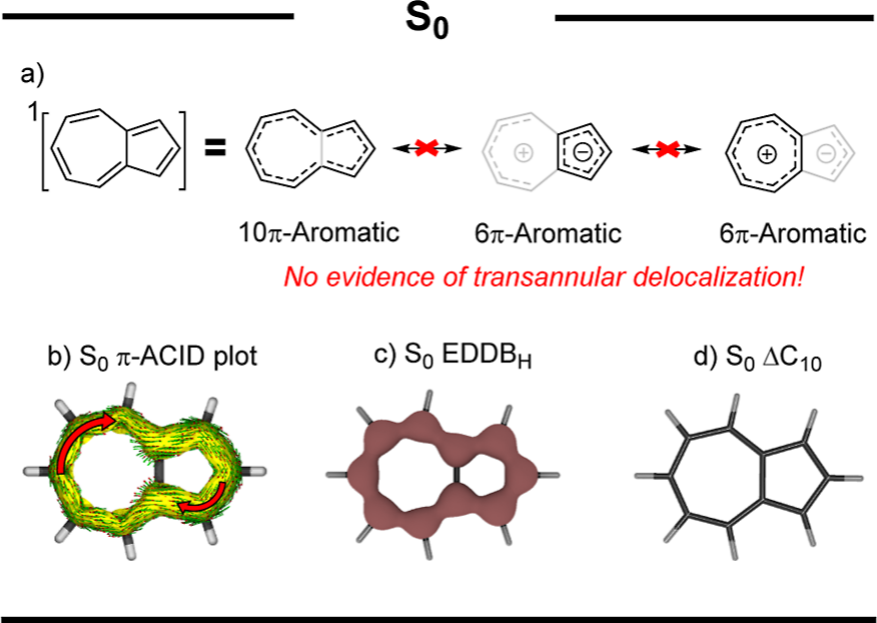
Summary of the results of S_0_ azulene; (a) important resonance structures, (b) S_0_ π-*A*CID plot (Figure S35), (c) S_0_ EDDB_H_, and (d) density of delocalized π-electrons in the transannular bond (Δ*C*_10_), which corresponds to the density of 0.02 electrons (amounting to virtually no delocalization through the transannular bond).

Ground state azulene is a Hückel 10π-aromatic molecule, as shown by all calculated aromaticity indices (Figure 2 and Chapter S5.1). Yet, only when comparing the calculated aromaticity indices of all possible delocalization circuits within its molecular geometry (Figure S6) do we find that the aromaticity of azulene originates from the delocalization along its perimeter. The calculated delocalization indices [aromatic fluctuation (FLU), multicenter delocalization (MCI), and electron density of delocalized bonds (EDDB)] indicate (Table S1) that most of the delocalized 10π-electron density is situated along the perimeter of azulene (cyclodecapentaenyl circuit). Conversely, delocalization circuits involving the transannular bond (cyclopentadienyl and cycloheptatrienyl) exhibit poor π-electron delocalization (Figure 2 and Table S1). By calculating EDDB_P_ electron counts (Table S2), we quantified the electron delocalization within the transannular bond of azulene (Δ*C*_10_). This bond exhibited only a negligible contribution, 0.02 electrons, to the global delocalized electron density of azulene (Figure 2). The virtual absence of ground-state transannular delocalization in azulene corresponds to its unusually long transannular bond of approximately 1.5 Å.^27^

The 10π-aromaticity of azulene was predicted in previous studies.^26,28^ However, the relevance of this finding has been largely overlooked. For example, the permanent dipole moment of azulene is usually explained by resonance structures that invoke delocalization through the transannular bond,^29^ but these resonance structures do not significantly contribute to the net electronic structure of azulene (Figure 2a), as evidenced by the negligible Δ*C*_10_ value. As a case in point, we found that homoazulene,^30^ the homoannelated counterpart of azulene without a conjugated transannular bond, has a similar permanent dipole moment (Chapter S13.1). Furthermore, in contrast to many other polycyclic aromatic hydrocarbons (PAH), such as the isoelectronic naphthalene (Chapter S13.2), which is known to favor the formation of multiple, 6π-aromatic rings,^31,32^ azulene’s S_0_ electronic structure resembles a single 10π-aromatic ring. Therefore, in the ground state, the bicyclic molecular geometry of azulene should be treated as a single 10π-aromatic hydrocarbon rather than a PAH.

**Figure 3:**
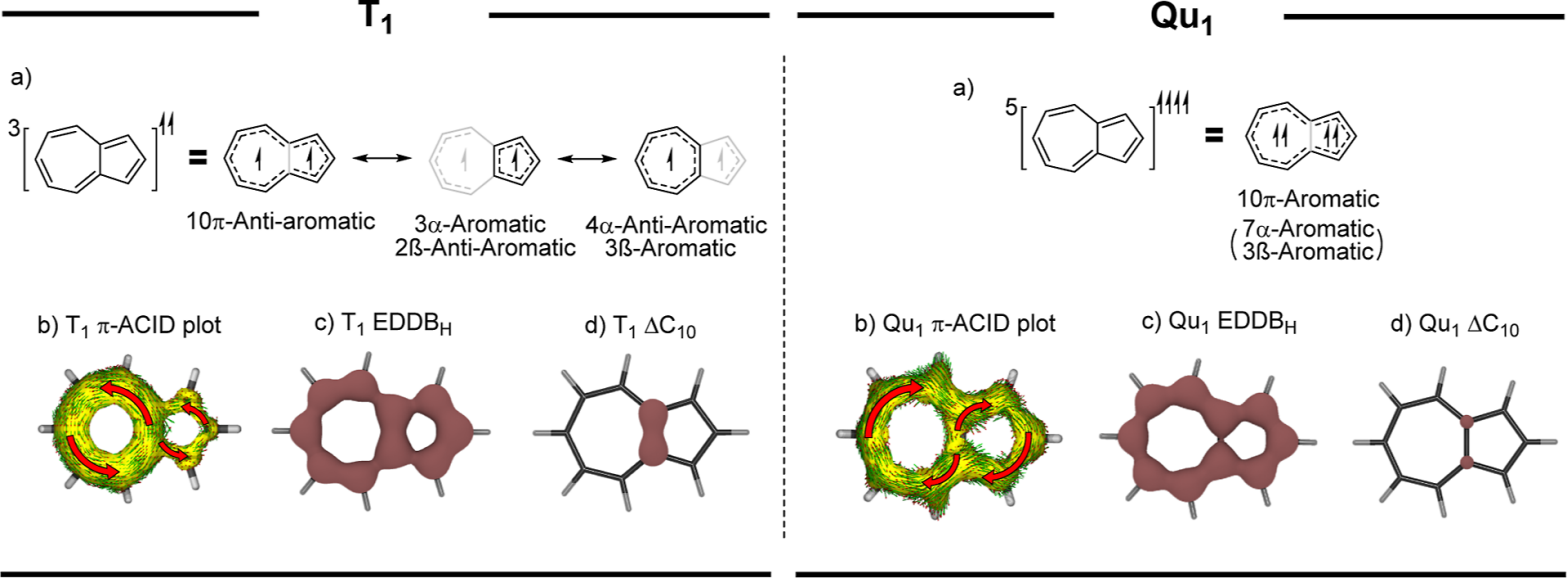
Summary of the results of T_1_ and Qu_1_ azulene; (a) most prevalent resonance structures, (b) π-*A*CID plots (Figures S36 and S37), (c) EDDB_H_, and (d) density of delocalized π-electrons in the transannular bond (Δ*C*_10_).

In the first triplet excited state, azulene follows Baird’s rules^19^ and is antiaromatic (Figure 3). This antiaromaticity is partly alleviated by transannular bond contraction. As a result, the delocalization decreases in the perimeter but increases in the cyclopentadienyl and cycloheptatrienyl circuits of azulene, as shown by our calculations (Table S5), and these circuits adopt the electronic structures of their corresponding cyclic radicals, as demonstrated by the EDDB (Chapter S12). The consequences of this enhanced geometric relaxation and the associated changes in the electronic structure of azulene can also be observed in the calculated isomerization stabilization energies (ISE) (Table S30), which are close to zero, or negative, for methylated isomers of T_1_ azulene. Despite the extensive reorganization and significant loss of antiaromaticity, T_1_ azulene remains, nevertheless, moderately antiaromatic.

The Qu_1_ of azulene has never been observed experimentally. However, previous theoretical studies have indicated that Qu_1_ azulene is aromatic.^25,26^ Furthermore, our results showed that the aromaticity of Qu_1_ originates primarily from the delocalization along its perimeter (Figure 3 and Table S9). The contrasting preferred electronic structures of T_1_ and Qu_1_ azulene are supported by both the perturbational molecular orbital (PMO) theory^28^ (Chapter S3.1.1) and Mandado’s rules^33^ (Chapter S3.1.2).

**Figure 4:**
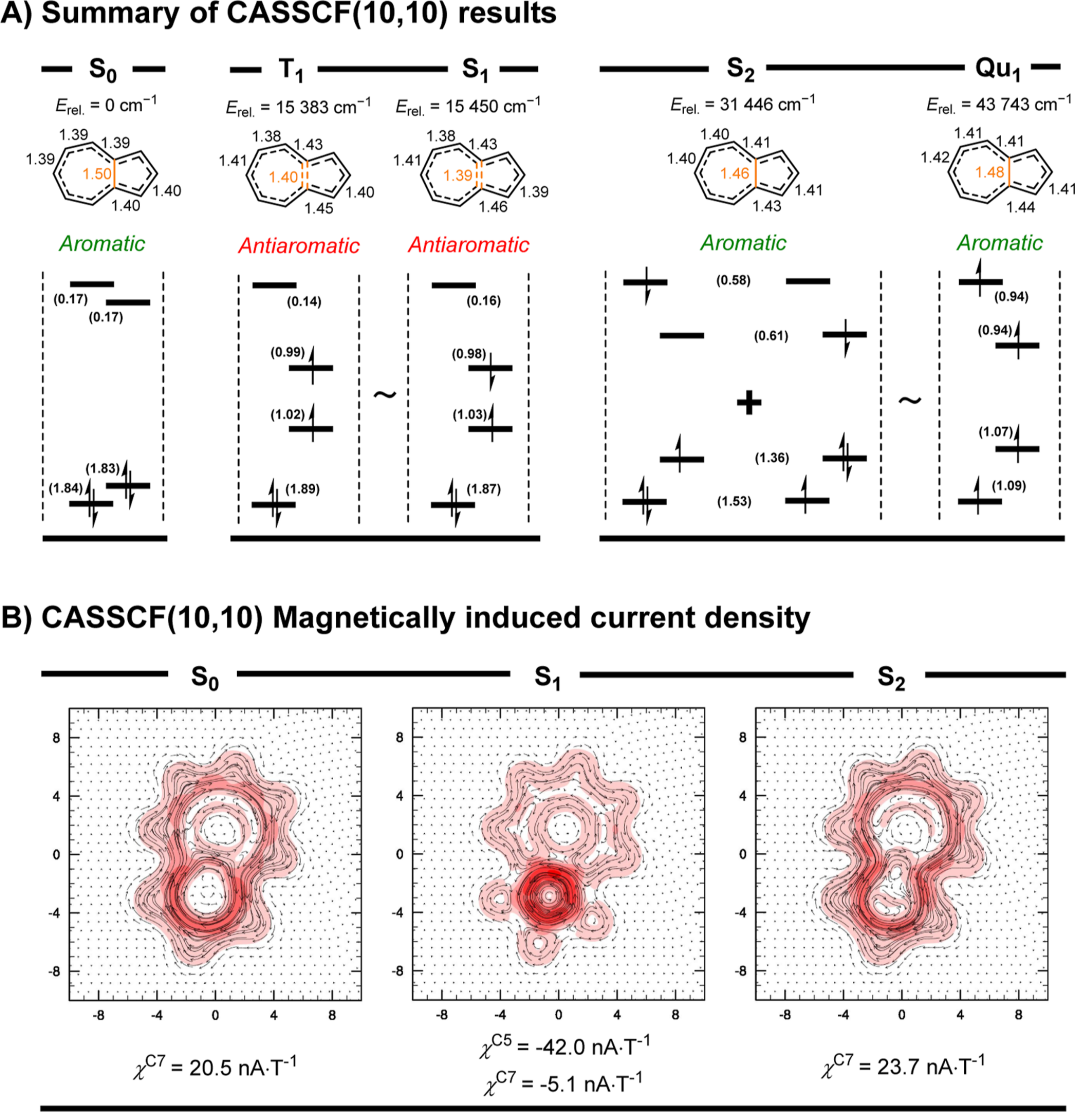
(A) Summary of calculated properties of the CASSCF(10,10) wave functions of the S_0_, S_1_, S_2_, T_1_, and Qu_1_ of azulene, grouped by their shared aromaticity (S_1_ ∼ T_1_, S_2_ ∼ Qu_1_), including their (from top): relative energies in reference to the S_0_ (*E*_rel._) which in case of S_1_ (318 nm) and S_2_ (647 nm) directly relate to the UV–vis absorption bands of azulene (see Figure 6B), bond lengths (*C*_2*V*_ symmetry), assigned aromatic character, canonicalized active-space natural MOs [reduced to (4,4) for clarity], their normalized occupancy (in brackets), and scheme of the dominant configuration(s). (B) CASSCF(10,10) MICD plot of azulene in S_0_, S_1_, and S_2_, constructed 1 au above the molecular plane (for full resolution plots, see Figures S38–S40), and the numerically integrated ring current susceptibilities of the cyclopentadienyl (χ^C5^) and cycloheptatrienyl (χ^C7^) rings (for integration planes, see Figure S7).

The concepts developed based on the S_0_, T_1_, and Qu_1_ azulene enabled us to evaluate the aromaticity of azulene in S_1_ and S_2_ (Figure 4). The complete active space self-consistent field (CASSCF) aromaticity indices (Table S13) indicated that the S_1_ azulene is antiaromatic, whereas its S_2_ is aromatic.

We investigated the (anti)aromaticity of S_1_ and S_2_ azulene further by calculating the magnetically induced current density (MICD) at the CASSCF(10,10) level.^34,35^ We found that azulene in S_2_, similarly to S_0_, exhibited a diatropic ring current along its perimeter. Conversely, azulene in S_1_ exhibited a paratropic ring current, localized primarily within the cyclopentadienyl and cycloheptatrienyl circuits (Figure 4B). The polarity of the MICD confirmed the antiaromaticity of azulene in S_1_ and the aromaticity in S_2_.

The S_1_ antiaromaticity of azulene followed Baird’s rules. Although Baird’s rules were originally formulated only for molecules in T_1_,^19^ in many molecules, the rules can be applied to S_1_ as well.^20^ The similarity between the S_1_ and T_1_ azulene is indicated by the calculated energies, minimum energy molecular geometries, aromaticity indices, and EDDB values (Figure 4 and Chapter S5.4).

Our findings also explain the aromaticity of S_2_ azulene. The root-optimized CASSCF(10,10) wave function of S_2_ azulene has a significant multireference character (Figure 4 and Tables S18 and S19). The wave function attributed near-degenerate occupancy by unpaired electrons to HOMO – 1 and LUMO and to HOMO and LUMO + 1. This factor plays a key role in the S_2_ aromaticity of azulene. The multireference character of S_2_ azulene mimics the Qu_1_ state by adopting a similar normalized π-orbital occupancy, in a relationship not unlike S_1_–T_1_. Consequently, the S_2_ and Qu_1_ states of azulene share a similar aromatic character.

In summary, S_1_ azulene is antiaromatic, and its geometry relaxes significantly to alleviate its antiaromaticity, whereas S_2_ azulene is aromatic, and its geometry does not relax significantly, thus preserving the energy gained upon excitation. Moreover, in antiaromatic S_1_, azulene adopts a biradical electronic structure. The biradical electronic structure of S_1_ azulene leads to spatial segregation of its two unpaired electrons into π and π* orbitals. Thus, in S_1_, azulene’s unpaired electrons exhibit low interelectron repulsion, which contributes to its low S_1_ energy (previously also described by Michl and Thulstrup).^36^ In the aromatic S_2_, conversely, the two unpaired electrons are delocalized within the azulene’s perimeter circuit. Accordingly, in S_2_ azulene, the unpaired electrons share a higher orbital overlap, resulting in higher interelectron repulsion. This contrast between the extent of geometric relaxation of azulene in S_1_ and S_2_ and the resulting difference in interelectron repulsion explains the large S_2_–S_1_ energy separation and, consequently, leads to a low rate of S_2_–S_1_ internal conversion (IC).

**Figure 5:**
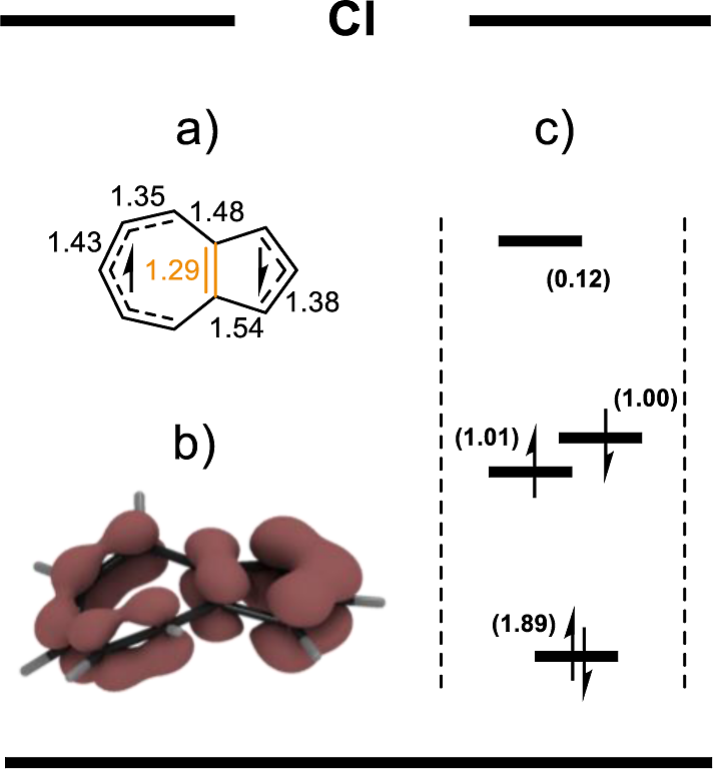
Summary of properties of the S_1_ CASSCF(10,10) wave functions of the S_1_–S_0_ conical intersection of azulene, calculated in the S_1_ state; (a) scheme of the proposed electronic structure and the bond lengths of CI azulene, (b) EDDB_H_ plot, (c) canonicalized active-space natural MOs [reduced to (4,4) for clarity], their normalized occupancy (in brackets), and scheme of the dominant configuration.

At this point, the absence of S_1_ emission caused by the depletion of S_1_ states via a S_1_–S_0_ conical intersection^18^ remained unaddressed though. To account for this key feature of the anti-Kasha behavior of azulene, we optimized the S_1_–S_0_ conical intersection geometry and calculated the CASSCF aromaticity indices (Section S5.5), as previously performed for S_1_ and S_2_. In the optimized conical intersection geometry, S_1_ azulene adopts the electronic structure of two acyclic radicals separated by a double bond (Figure 5), in turn increasing the overall energy. This increase is offset by subsequent nonradiative transition to the aromatic ground state (Table S1). As suggested by canonicalized active-space natural MO’s (Figure 5c), at the CI geometry, azulene exhibits low HOMO–LUMO separation. This low separation favors the pairing of its two unpaired electrons (in S_1_), thus providing a nonradiative pathway to S_0_. Therefore, the conical intersection enables S_1_ antiaromaticity relief.

## 3 Conclusions

**Figure 6:**
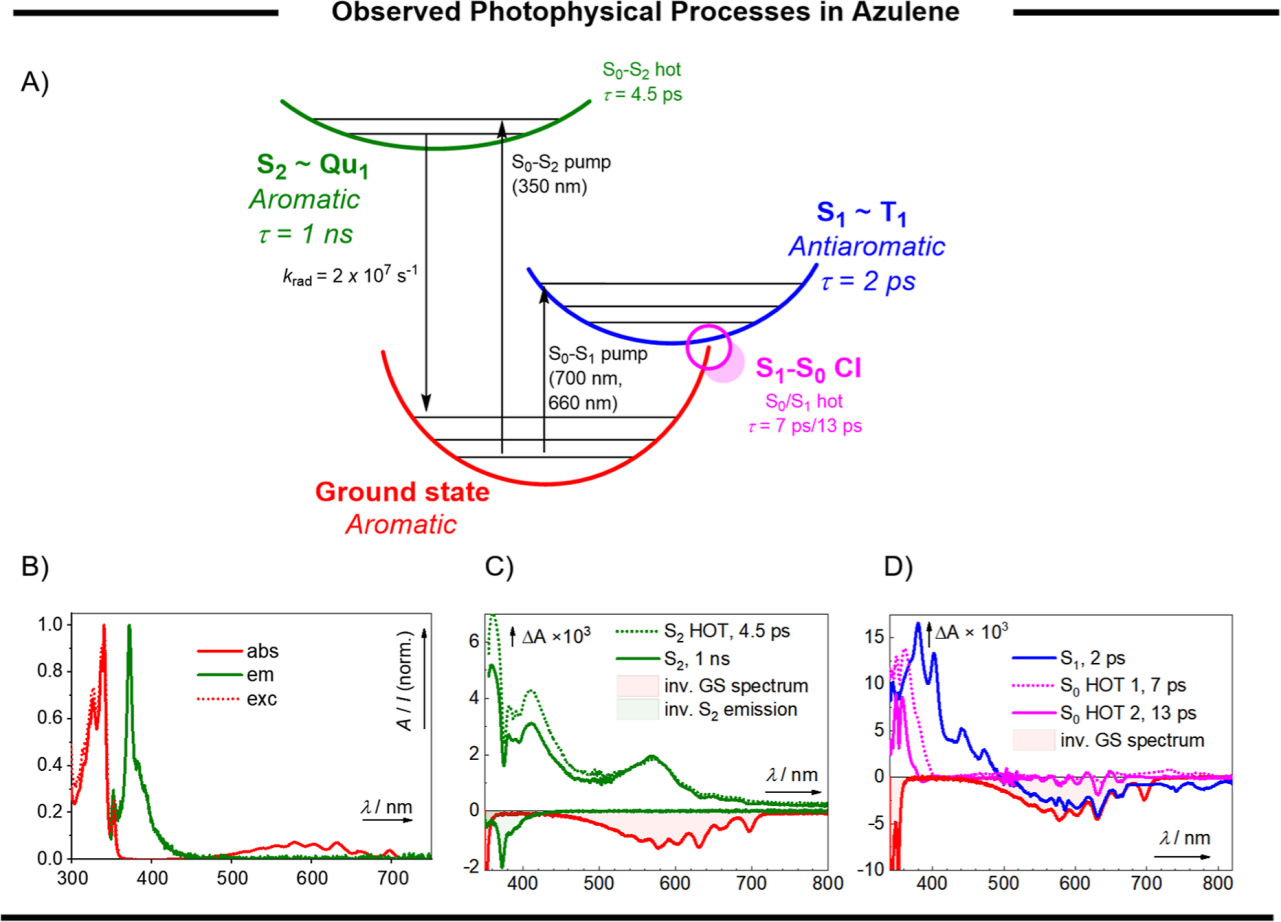
(Anti)aromaticity of the lowest three singlet states of azulene (S_0_, S_1_, and S_2_) explains its anti-Kasha behavior. (A) Jablonski diagram of the experimentally observed photophysical phenomena, (B) UV–vis absorption, emission (λ_exc_ = 338 nm), and excitation spectra (λ_em_ = 372 nm) of azulene, (C) species associated spectra (SAS) of azulene excited at 350 nm (*E* = 600 nJ), and (D) SAS of azulene excited at 700 nm (*E* = 2 μJ). All spectra were recorded in cyclohexane.

In conclusion, the (anti)aromaticity of the lowest three singlet states of azulene (S_0_, S_1_, and S_2_) explains its anti-Kasha behavior. Azulene is aromatic in its ground state, antiaromatic in its S_1_, and aromatic in the S_2_. The (anti)aromaticity of each state matches its lifetime, as experimentally determined by transient absorption spectroscopy (Figure 6 and Chapter S11). Moreover, the S_1_ azulene’s geometry relaxes significantly to alleviate its antiaromaticity. Consequently, the S_1_ minimum-energy geometry of azulene is found near a conical intersection.

In the antiaromatic, S_1_ minimum-energy geometry, azulene readily undergoes a favorable, nonradiative transition to its aromatic ground state through a conical intersection (Figure 6A). The depletion of S_1_ through the conical intersection provides unimolecular antiaromaticity relief. By contrast, the aromatic S_2_ is stabilized, does not undergo significant geometric relaxation, and maintains at high energy, which causes a low rate of S_2_–S_1_ IC. For this reason, the S_2_ state of azulene is long-lived and emitting, breaking Kasha’s rule.

## 4 Methods

Both PMO analysis^28^ and Mandado’s rules,^33^ wherein π-electrons are separated by their spin (*m*), were extensively used in this study and supported by quantum chemical calculations of delocalization and aromaticity indices. We calculated HOMA,^37,38^ MCI,^39^ and FLU^40^ and *I*_ring_(41) indices for the C_5_, C_7_, and C_10_ circuits (Figure S6); in both “net” and “spin-separated” formulations, for all above states. We used the EDDB scheme, which represents electron delocalization that cannot be assigned to atoms or bonds due to its (multicenter) delocalized nature, to integrate the number of globally (EDDB_G_ and EDDB_H_) and locally (EDDB_F_, EDDB_E_, and EDDB_P_) delocalized π-electrons^42,43^ and, in particular, the density of delocalized π-electrons in the transannular bond of azulene (Δ*C*_10_). Note that the spin-separated values of each index can be interpreted in line with Mandado’s rules in open-shell systems. We also calculated the NICS^44^ at the centroid of C_5_ and C_7_ fragments and constructed ACID^45^ plots of S_0_, T_1_, and Qu_1_ azulene and MRSCF MICD at CASSCF(10,10) level for S_0_, S_1_, and S_2_.^34,35^ ISE of methylated derivatives of S_0_ and T_1_ azulene were also calculated.^46^ Full details on the computational methods and theoretical approach and the measured stationary absorption and emission and transient absorption spectra are provided in the Supporting Information.

## Supporting Information Available

PMO theory and Mandado’s rules, computational methods, ground and excited state aromaticity calculations, S_1_–S_0_ conical intersection, NICS, dipole moment, ISE, ACID, MICD calculations, absorption and emission spectroscopy, time-resolved spectroscopy, and azulene versus naphthalene and homoazulene (PDF)
Optimized geometries (ZIP)
