# Supplementary material for: Excited-State (Anti)Aromaticity Explains Why Azulene Disobeys Kasha’s Rule
Source: J Am Chem Soc. 2023 Sep 13;145(39):21569–75. doi: 10.1021/jacs.3c07625 (PMC10557139; doi:10.1021/jacs.3c07625)
Supplement: Supplementary file 1 — ja3c07625_si_001.pdf [file ja3c07625_si_001.pdf]

SUPPORTING INFORMATION

Excited-State (Anti)Aromaticity Explains Why Azulene Disobeys Kasha's Rule

David Dunlop [a,b] *Lucie Ludvíková,* [a] *Ambar Banerjee,* [c] *Henrik Ottosson,* [d,]* *and Tomáš Slanina,* [a,]*

[a] Institute of Organic Chemistry and Biochemistry of the Czech Academy of Sciences, Flemingovo náměstí 542/2, Prague 6, 160 00 Czech Republic

[b] Department of Inorganic Chemistry, Faculty of Science, Charles University in Prague, Hlavova 2030, 128 40 Praha 2, Czech Republic

[c] Division of X-ray Photon Science, Department of Physics and Astronomy - Ångström Laboratory, Uppsala University, Box 516, Uppsala 751 20, Sweden

[d] Department of Chemistry - Ångström Laboratory, Uppsala University, Box 523, Uppsala 751 20, Sweden

Contents:

S1 Author's contributions 3

S2 Materials and methods 4

S3 Theoretical approach: PMO theory and Mandado's rules 5

S3.1 (Anti)Aromaticity of azulene in S_0, T_1 and Qu_1 states evaluated using the PMO theory and Mandado's rules 7

S3.1.1 (Anti)Aromaticity of S_0, T_1 and Qu_1 azulene according to the PMO theory 7

S3.1.2 (Anti)Aromaticity of S_0, T_1 and Qu_1 azulene as per Mandado's rules 10

S4 Computational methods 11

S5 Computational results: Aromaticity of azulene 13

S5.1 Ground state (S_0) azulene 13

S5.2 First triplet excited state (T_1) azulene 17

S5.3 First quintet excited state (Qu_1) azulene 21

S5.4 First excited singlet (S_1) and second excited singlet (S_2) state azulene 24

S5.5 S_1-S_0 conical intersection 30

S6 Computational results: Functional dependency of calculated aromaticity indices 35

S7 Computational results: NICS-xy and NICS-z scan 38

S8 Computational results: Dipole moment of azulene in S_0, T_1 and Qu_1 states 41

S9 Computational results: Isomerization stabilization energy (ISE) of azulene in the S_0 and T_1 states 42

S10 Experimental results: Absorption and emission spectrum of azulene 44

S11 Experimental results: Time-resolved spectroscopy of azulene 45

S12 Extended discussion: Electronic structure of T_1 azulene compared to that of cyclopentadienyl and cycloheptatrienyl radicals 49

S13 Extended discussion: Azulene compared to naphthalene and homoazulene 50
S13.1 Homoazulene 50
S13.2 Naphtalene 53
S14 Full-resolution π-ACID plots 54
S15 Full resolution MICD plots 57
S16 Coordinates in .XYZ format 60
S17 References 61

S1 Author's contributions

D.D conceived the research hypothesis, with inputs from H.O. and T.S. H.O., T.S. and D.D. conceptualized the research methods. D.D. performed the quantum chemical computations, A.B. contributed by the calculation of CASSCF(10,10) MICD. L.L. performed the experimental investigation. D.D wrote the paper, with inputs from all co-authors.

S2 Materials and methods

Azulene was purchased from Merck (Germany). Solvents were purchased from Acros Organics (part of Thermo Fisher Scientific, Belgium).

UV-Vis absorption (Agilent Cary 8454 UV-Vis) and UV-Vis emission spectra (Horiba Duetta spectrofluorometer) were recorded at room temperature. Transient absorption experiments were carried out using commercially available apparatus from Ultrafast Systems. Briefly, an Ytterbium femtosecond laser was used to generate 1030 nm with 400 μJ pulses at 1 kHz. The output was split into two parts, where 75% of the 1030-nm pulses were used to pump a collinear Optical Parametric Amplifier (OPA, APOLLO-Y) tuned to pump 350, 660 or 700-nm pulses (ca. 250 fs, 600 - 2000 nJ at the sample position) for sample excitation and 25% were used for generation of supercontinuum white light probe pulses by focusing into one of two different crystals giving a probe spectrum ranging from 350 – 500 or 500 – 850nm. Probe pulses were delayed via an optical motorized delay line with 8 ns time window. The pump and probe pulses were focused colinearly into the sample to spot sizes of ca. 250 and 100 μm full width at half maximum, respectively. The spectrometer used a linear array detector with CMOS sensor (1024 pixel). The isotropic spectral signals were secured by use of an achromatic broadband depolarizer for the pump (ThorLabs, DPP-25-A). The data were corrected for chirp. The sample in cuvette with 2 mm pathlength was randomly moved at 1 mm/s speed through the measurement. The stability of the sample was verified by recording steady-state absorption spectra before and after each measurement.

S3 Theoretical approach: PMO theory and Mandado's rules

Excited-state (anti)aromaticity was derived by Colin Baird for the lowest ππ* triplet state, using the perturbational molecular orbital (PMO) theory.[1-2] In the ground-state PMO analysis of cyclic π-conjugated molecules, one first divides the molecules into two radical fragments with an odd number of carbon atoms.[1] Based on orbital symmetry, one then defines the molecular orbitals (MOs, singly occupied = SOMOs) of the two fragments that would interact if the fragments were unified. One considers the unification of the two fragments as a small perturbation, which enables them to analyse the resulting changes in molecular orbital energies using perturbational theory equations and to qualitatively assess whether the unification of the two molecular fragments lowered the orbital energies of each MO. Based on the net result, one can determine whether the resulting molecule has a stabilizing or destabilizing electron configuration.

Baird used the PMO approach, but assumed that unifying two radical fragments would form a molecule of triplet multiplicity.[2] He then identified two key orbital interactions between the fragments of a triplet state molecule (i) type I (a destabilizing interaction of two SOMO with the same symmetry) and (ii) type II (a stabilizing interaction of SOMO with doubly-occupied or unoccupied MOs of the same symmetry) interactions. Summarizing the outcomes of his analysis as rules based on π-electron density, he stated that molecules with 4*n* π-electrons are stabilized in their T_1 – aromatic – state, whereas molecules with (4*n*+2)π-electrons are destabilized in their T_1 – antiaromatic – state. Baird's rules for ππ* excited-state (*anti*)aromaticity exactly contrast with Hückel's rules for ground state aromaticity (Figure S1).

More universal rules were established by Mandado and colleagues.[3] Mandado's rules are based on the density of π-electrons separated by their spin (*m*); an odd *m*-electron density (2*n*+1) yields aromatic and an even *m*-electron density (2*n*) yields antiaromatic contributions to the global aromaticity of a molecule (*n* = 0, 1, 2, 3…), resulting in net aromatic, net antiaromatic and mixed aromatic/antiaromatic π-conjugated cyclic molecules and radicals.

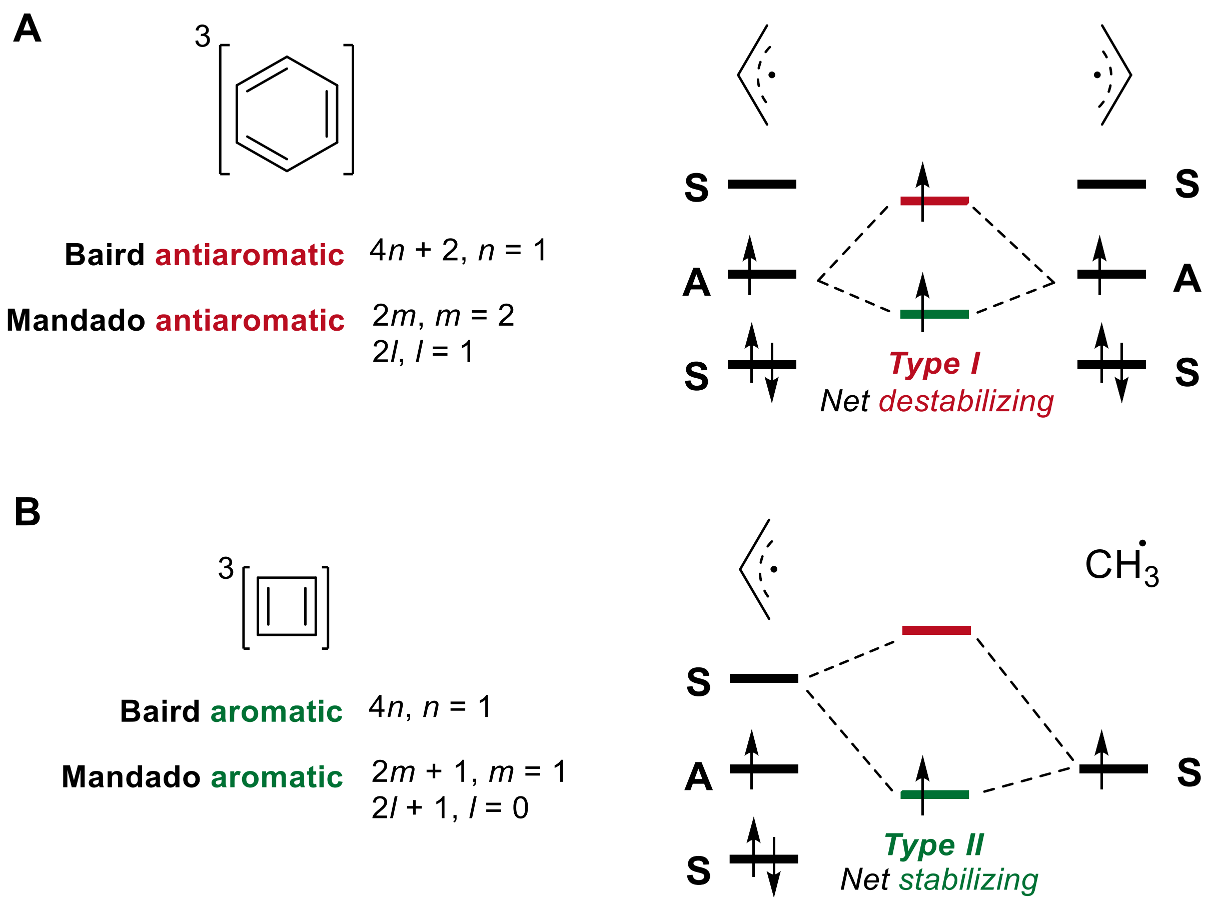

Figure S1: Left: A) Benzene and B) cyclobutadiene, along with Baird's and Mandado's rules-derived aromaticity in T_1 state; Right: T_1 PMO decomposition of A) benzene and B) cyclobutadiene, highlighting the destabilizing Type I and stabilizing Type II interaction. All destabilizing factors are highlighted in red, and all stabilizing factors are highlighted in green.

S3.1 (Anti)Aromaticity of azulene in S_0, T_1 and Qu_1 states evaluated using the PMO theory and Mandado's rules

We qualitatively evaluated the aromaticity of azulene using the PMO theory and Mandado's rules (Figure S2-Figure S5). We used the Hückel theory to evaluate the energies of the radicals in PMO diagrams. Our PMO diagrams were plotted to highlight only the most impactful interactions – those of the singly occupied molecular orbitals (SOMO) of the acyclic radicals. Conversely, Mandado's rules required only considering the possible resonance structures of azulenes in the studied states.

S3.1.1 (Anti)Aromaticity of S_0, T_1 and Qu_1 azulene according to the PMO theory

In this study, we showed that the PMO theory can be used to qualitatively rationalize not only the S_0 but also the T_1 and Qu_1 properties of azulene. Moreover, beyond the derivation of ground and T_1 state aromaticity of small annulenes, the PMO theory can also be used to determine the favoured delocalization circuits in polycyclic conjugated compounds in excited states and to provide a rational explanation for the aromaticity of the Qu_1 state.

The S_0 perimeter aromaticity of azulene was first hinted at by Dewar.[1] Any combination of odd-membered acyclic radicals with the combined number of 10 carbons will result in a stabilizing ground-state electron configuration, as the SOMO of these two radicals will always result in a symmetric union (Figure S2).

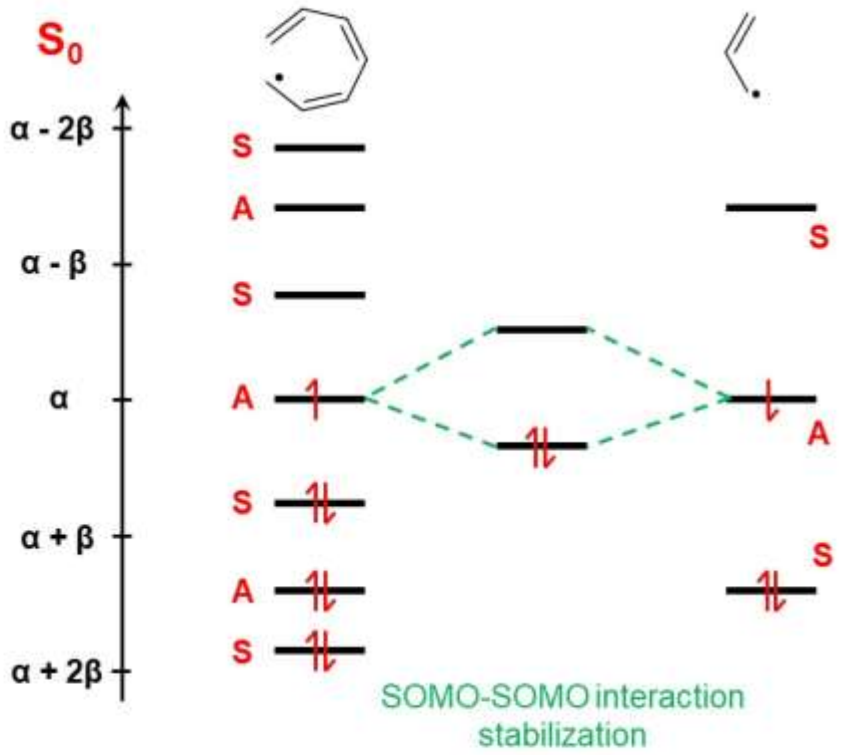

Figure S2: PMO diagram of S_0 azulene as a product of an interaction between C_7 and C_3 linear radicals. Molecular orbitals are assigned S or A symbols based on their orbital symmetry, in reference to their σ_v mirror planes.

The aromaticity of azulene is caused by the stabilizing interaction of the SOMOs. However, as found by Baird, the same symmetric union results in a destabilizing, type I interaction in the T_1 state (Figure S3).

Nevertheless, we propose that the perimeter antiaromatic electronic structure is in competition with yet another electronic structure which does not exhibit type I interactions – the electronic structure of cyclic radicals, which should be stabilized in the T_1 state as per our analysis. Although this application extends beyond the scope of Baird's approach,[2] the conditions of Dewar's PMO theory from which excited-state aromaticity was derived, in principle, allows such treatment. This analysis provided a rational explanation for the observed electronic structure of the T_1 azulene, which shows enhanced transannular delocalization, in line with all calculated aromaticity indices (Table S5, Table S1).

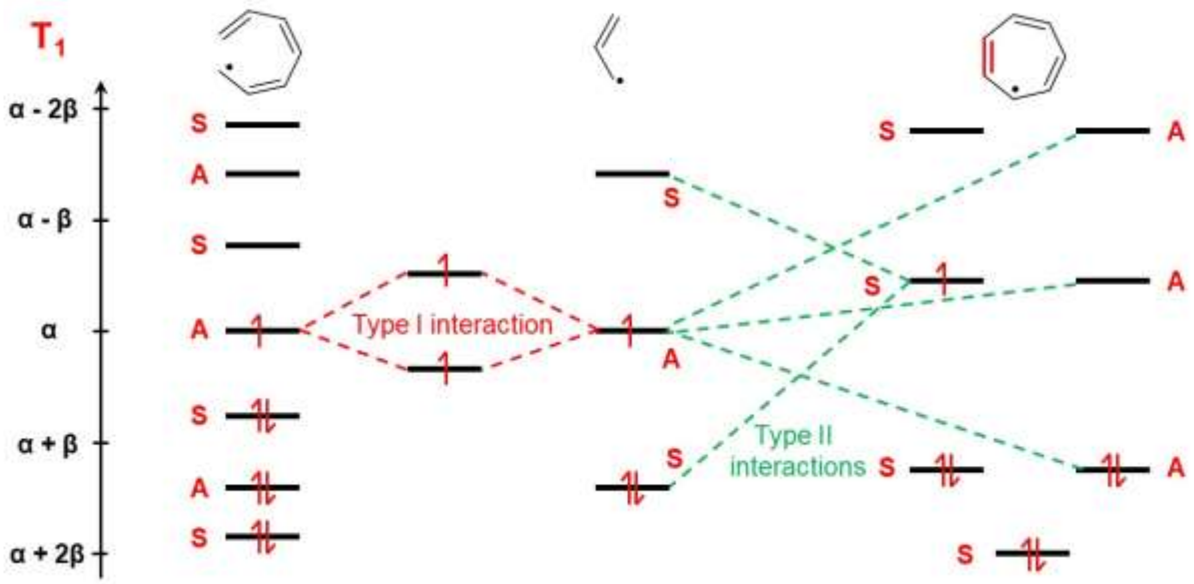

Figure S3: PMO diagram of T_1 azulene, as a product of interactions between C_7 and C_3 (left) linear radicals, and (right) between a C_3 linear and C_7 cyclic radical. Orbitals are assigned S or A symbols based on their orbital symmetry, σ_v mirror planes. We highlight the Type I interaction (red) on the left side as the cause of the perimeter antiaromaticity of T_1 azulene. Conversely, on the right side, type I interaction is absent, and only the Type II interactions (green) of SOMOs and other stabilizing, doubly occupied orbitals of the same symmetry are highlighted.

Lastly, we examined whether the PMO theory can be used to rationalize the previously reported aromaticity of Qu_1 azulene (Figure S4).[4] By comparing the interaction of the allyl radical with both linear and cyclic C_7 triradicals, we found that, regardless of orbital symmetry, the resulting orbital occupancy of Qu_1 azulene will leave the antibonding orbitals φ*, which lead to a destabilizing type I interaction, empty. The sum of the orbital energies of the electrons in unified fragments is thus lower than the sum in each individual fragment. Therefore, Qu_1 azulene may be perimeter aromatic. Furthermore, transannular delocalization is also stabilizing within the constructed PMO model.

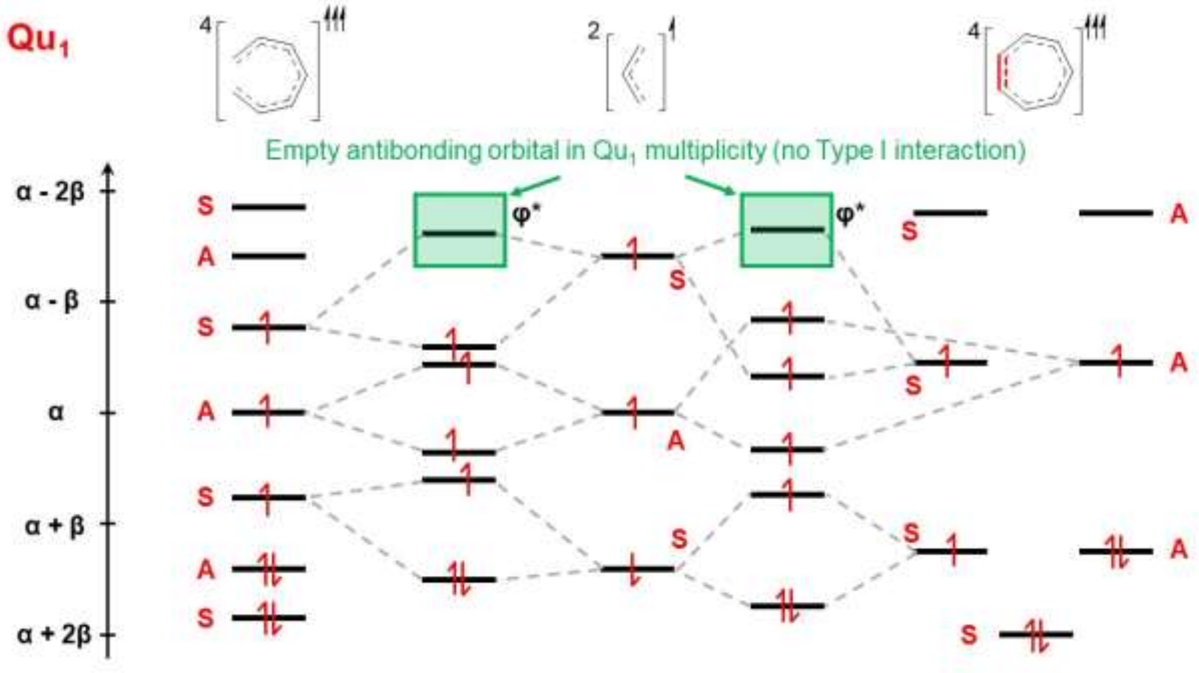

Figure S4: PMO diagram of Qu_1 azulene as a product of interactions between C_7 linear radical triradicals and C_3 linear radicals (left) and between C_3 linear radicals and C_7 cyclic triradicals. Orbitals are assigned S or A symbols based on their orbital symmetry, in reference to their σ_v mirror planes. We highlight the empty (would-be) destabilizing orbitals φ* of Qu_1 azulene (green) as the cause of its net aromaticity.

S3.1.2 (Anti)Aromaticity of S_0, T_1 and Qu_1 azulene as per Mandado's rules

Mandado's rules provide a simpler explanation for the excited state anti(aromaticity) of azulene, in line with the results of the PMO theory.[3, 5] A schematic representation summarising the application of Mandado's rules to azulene is shown below (Figure S5). Equivalent resonance structures are disregarded. Note that Mandado's rules correlate with our calculated spin-separated aromaticity indices (Table S5, Table S9).

Please note that Mandado's rules do not quantify the magnitude of (anti)aromatic contributions in open-shell molecules. Thus, the net aromaticity of electronic configurations with e.g. α-aromatic/β-antiaromatic character must be determined by calculating global aromaticity indices.

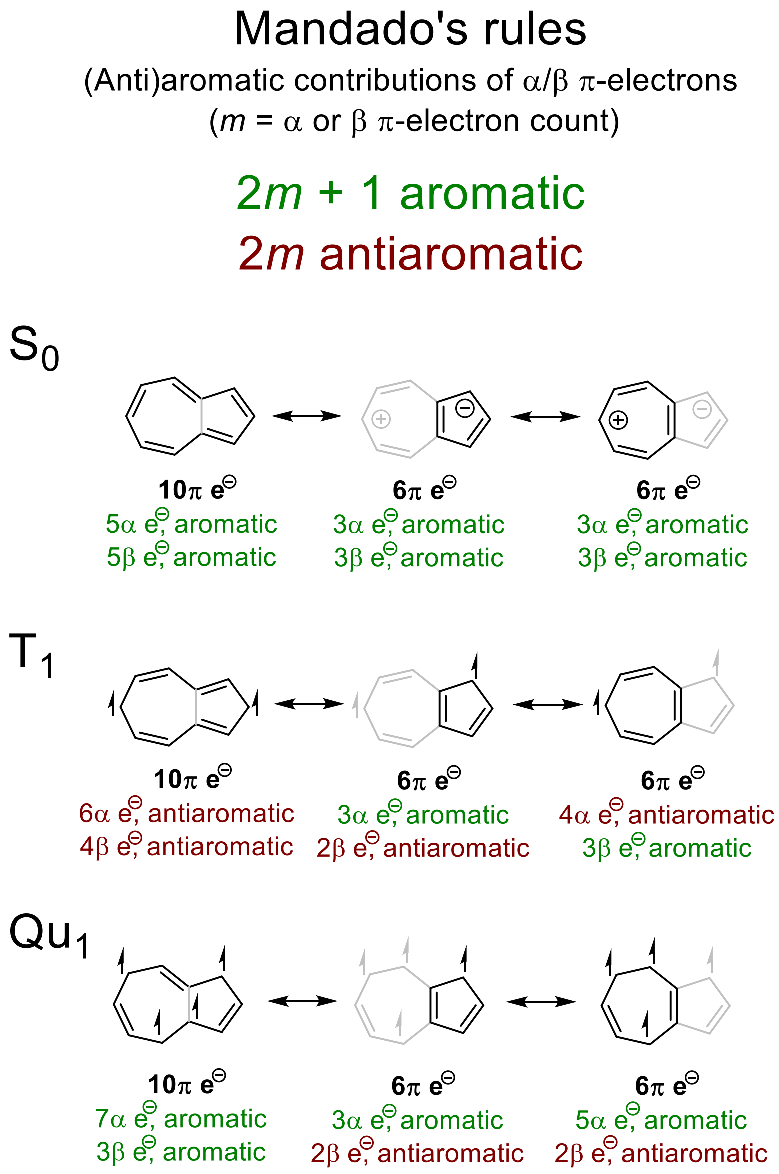

Figure S5: A summary of Mandado's rules applied to S_0, T_1 and Qu_1 azulene and its (non-redundant) resonance structures.

S4 Computational methods

Quantum chemical calculations were performed using the ORCA 5.0.3,[6] Gaussian 9[7] and Dalton2011 and Dalton2016.2 software packages.[8] Conformers of each compound were screened in CREST 2.12.[9] EDDB plots were calculated using the June 26th 2021 edition of the EDDB script, [10] on wavefunctions diagonalized by NBO 7.0.[11] Delocalization indices (MCI, I_{ring}) were calculated using the AIMAll[12] and ESI-3D[13-14] packages.

DFT (RKS and UKS) optimizations, single-point calculations and time-dependent calculations applied a def2-TZVPP basis set,[15] Grimme's D3 dispersion correction with Becke-Johnson damping.[16-17] Orca calculations employed a RIJCOSX[18] density fitting scheme (def2/J auxiliary basis set, 'defgrid2' integration grid, 'verytighscf' and 'tightopt' constraints. Geometry optimizations were performed on GFN2-xtb[19] preoptimized geometries (only a single conformer was found for azulene). Ground state geometries of the studied compounds (where relevant, both in neutral and protonated state) were optimized using the (U)B3LYP exchange-correlation functional. Gradients at TD-B3LYP level were applied to optimize excited state singlet minimum energy geometries. The procedures were repeated using (U)BLYP and (U)CAM-B3LYP[20] exchange-correlation functionals in selected cases to compare the results of aromaticity indices. Both ground state and excited state minimum energy geometries were verified by vibrational frequency calculations (applying analytical Hessian in the ground state and numerical Hessian in the excited state) at constraints corresponding to geometry optimization. An optimization of the S_1-S_0 conical intersection was performed at the TD-B3LYP level, starting from S_1 minimum-energy geometry and applying first-order non-adiabatic coupling matrix elements,[21] and electron translation factors.[22]

Single-point CASSCF(10,10)/def2-TZVPP wavefunctions were calculated using Orca 5.0.3 software package. The calculations utilized (U)MP2 wavefunction calculated at optimized geometries as the starting guess. No-iteration runs were performed and thereafter, active space was rotated to contain azulene's 5π and 5π* orbitals. Then, full CASSCF(10,10)/def2-TZVPP run with NEVPT2 dynamic correlation treatment (using the fully internally contracted, DLPNO approach)[23] was performed. For S_1 and S_2 states, root-optimized wavefunctions were converged. Conical intersection geometry was evaluated at both S_0 and S_1 configuration.

RKS and UKS NICS indices were calculated using Orca 5.0.3 for S_0, T_1, and Qu_1 azulene at (U)B3LYP/6-311+G** level of theory. CASSCF(10,10)/6-311+G** NICS indices were calculated using Dalton2016.2.

ISE were calculated using ORCA 5.0.3. Geometries of isomers were optimized at (U)B3LYP/def2TZVPP level of theory. Then, an additional single point (U)-DLPNO-CCSD(T)/def2-TZVPP calculation[24-25] was performed.

ACID plots were calculated using Gaussian 9 software package.

The magnetically induced current density (MICD) for the azulene molecule was computed at the CASSCF(10,10)/TZVP level of theory as implemented in Dalton 2011 code.[26-28] The current densities were obtained by first-order response calculations using gauge-including atomic orbitals (GIAOs) in the CASSCF(10,10) framework. The perturbation operator was taken to be a magnetic dipole operator oriented perpendicular to the molecular plane. The excited states, S_1 and S_2 state, were specifically and individually optimized in the CASSCF framework. The 2-dimensional Gauss–Lobatto quadrature grid was used to integrate the ring current susceptibilities.[29] The plane of integration was chosen to be perpendicular to the molecular plane, extending 10 Bohr outward from the center of the ring, from 5 Bohr above to 5 Bohr below (Figure S7).

A) Fragmentation scheme

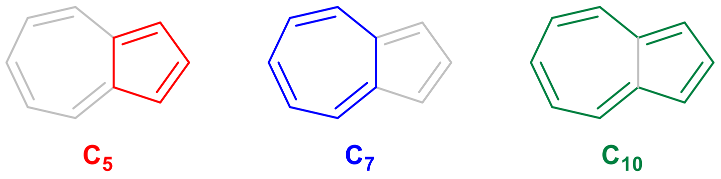

B) ΔC_{10} $EDDB_P$ Subtraction Scheme

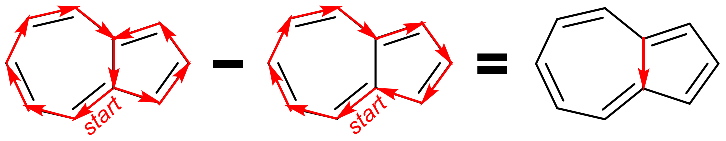

Figure S6: Fragmentation and subtraction schemes used in the calculation of aromaticity indices. In B, the $EDDB_P$ index integrates the "local" electron density delocalized in bonds, over a defined pathway (per our definition, leading to a ΔC_{10} value quantifying the electronic delocalization across the transannular bond).

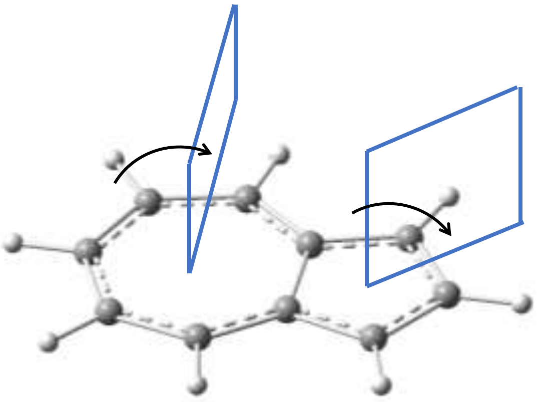

Figure S7: The half integration planes (blue) used for the calculation of the current susceptibilities, across the cyclopentadienyl and cycloheptatrienyl rings of azulene. The black arrows depict the direction of diatropic ring currents in an aromatic scenario.

S5 Computational results: Aromaticity of azulene

S5.1 Ground state (S_0) azulene

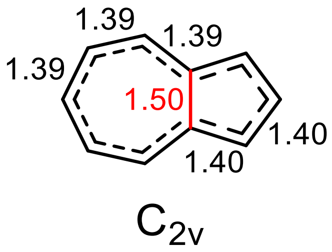

Figure S8: Summary of bond lengths of S_0 azulene in its optimized minimum-energy geometry (point group C_{2v}) B3LYP/def2-TZVPP.

Table S1: Summary of aromaticity indices for S_0 azulene in its optimized minimum-energy geometry, harmonic oscillator model of aromaticity (HOMA), ring-size normalized multicenter bond index (MCI), aromatic fluctuation index (FLU), ring-size normalized Giambagi multicenter index (I_{ring}) and zz component of the nucleus independent chemical shift 1.7 Å above the centre of the ring ($NICS_{zz}(1.7)$), calculated at B3LYP/6-311+G**(RKS), and CASSCF(10,10)/def2-TZVPP for MCI and CASSCF(10,10)/6-311+G** for NICS. Rings C_5, C_7 and C_{10} assigned per scheme in Figure S6.

Index	Ring	RKS	CAS-SCF
**HOMA**[a]	C_5	0.37	[a]
	C_7	0.57	[a]
	C_{10}	0.98	[a]
**$MCI^{1/n}$**	C_5	0.460	0.408
	C_7	0.557	0.501
	C_{10}	0.647	0.581
**FLU**	C_5	0.024	[b]
	C_7	0.017	[b]
	C_{10}	0.009	[b]
**$I_{ring}^{1/n}$**	C_5	0.471	0.426
	C_7	0.523	0.476
	C_{10}	0.570	0.526
**$NICS_{zz}(1.7)$**	C_5	-30.1	-26.7
	C_7	-19.1	-16.4

[a] Geometric index; without using a calculated wavefunction.
[b] Current software implementation is not compatible with multireference methods.

Table S2: EDDB electron densities of S_0 azulene in its optimized minimum-energy geometry at B3LYP/def2-TZVPP level of theory; $EDDB_G$ – density of globally delocalized electrons in bonds, $EDDB_H$ – density of globally delocalized electrons in bonds of heavy atoms, $EDDB_F$ – density of delocalized bonds in bonds of fragments (local contributions), $EDDB_E$ – density of delocalized electrons in bonds of fragments (local+non-local contributions), $EDDB_P$ – density of delocalized electrons in bonds along a pathway. Rings C_5, C_7 and C_{10} assigned per scheme in Figure S6.

Index	Fragment/path	Electron density
**$EDDB_G$**	-	11.0
**$EDDB_H$**	-	10.3
**$EDDB_F$**	C_5	2.6
	C_7	4.5
**$EDDB_E$**	C_5	5.5
	C_7	7.5
**$EDDB_P$**	C_5	2.4
	C_7	4.0
	C_{10}(perimeter)	8.3
	C_{10}(transannular)	8.3

Table S3: Active space orbital energies and normalized densities of S_0 azulene, calculated at CASSCF(10,10)/def2-TVPP.

Active MO	Orbital energy (eV)	Normalized density
1	-14.05	1.96
2	-11.87	1.94
3	-10.56	1.94
4	-7.74	1.84
5	-6.71	1.83
6	2.41	0.17
7	1.59	0.17
8	8.09	0.06
9	10.67	0.05
10	10.28	0.05

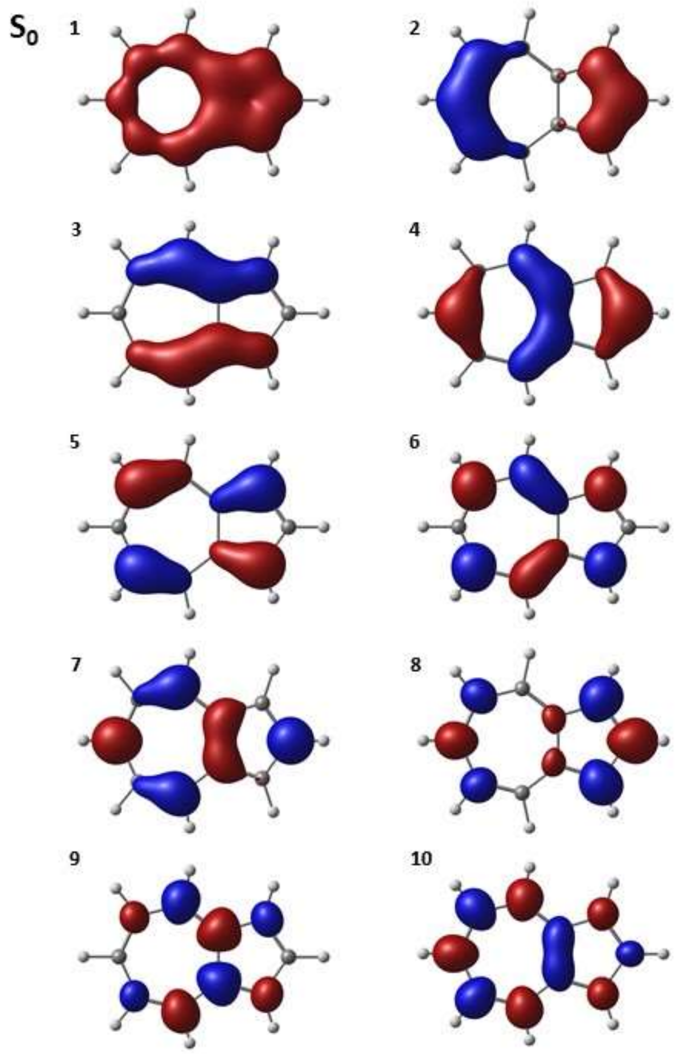

Figure S9: Active MOs of S_0 azulene, calculated at CASSCF(10,10)/def2-TVPP.

Table S4: CI vector of the S_0 azulene, calculated at CASSCF(10,10)/def2-TVPP (configuration printed as a string of orbital occupancies, in decreasing order of active MOs).

Configuration	Weight
2222200000	0.772
2221111000	0.043
2222020000	0.029
2220202000	0.028
2121201100	0.011
2122110100	0.011
2212110010	0.009
2211201010	0.008
1221201001	0.007
1222110001	0.006

S5.2 First triplet excited state (T_1) azulene

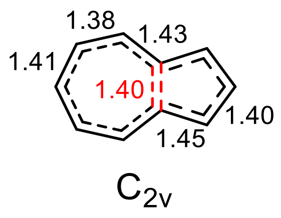

Figure S10: Bond lengths of T_1 azulene in its optimized minimum-energy geometry (point group C_{2v}) at U-B3LYP/def2-TZVPP level of theory.

Table S5: Summary of aromaticity indices for T_1 azulene in its optimized minimum-energy geometry, harmonic oscillator model of aromaticity (HOMA), ring-size normalized multicenter bond index (MCI), aromatic fluctuation index (FLU), ring-size normalized Giambagi multicenter index (I_{ring}) and zz component of the nucleus independent chemical shift 1.7 Å above the centre of the ring ($NICS_{zz}(1.7)$), calculated at UB3LYP/6-311+G** (UKS) , and CASSCF(10,10)/def2-TZVPP for MCI and CASSCF(10,10)/6-311+G**for NICS. Rings C_5, C_7 and C_{10} assigned per scheme in Figure S6.

Index	Ring	UKS(net)	UKS(α)	UKS(β)	CAS-SCF
**HOMA**	C_5	0.55	[a]	[a]	[a]
	C_7	0.85	[a]	[a]	[a]
	C_{10}	0.67	[a]	[a]	[a]
**$MCI^{1/n}$**	C_5	0.503	0.488	0.338	0.415
	C_7	0.579	0.343	0.576	0.488
	C_{10}	-0.584	-0.490	-0.573	-0.369
**FLU**	C_5	0.015	0.003	0.033	[b]
	C_7	0.007	0.018	0.003	[b]
	C_{10}	0.012	0.014	0.017	[b]
**$I_{ring}^{1/n}$**	C_5	0.484	0.458	0.364	0.438
	C_7	0.533	0.400	0.522	0.476
	C_{10}	0.473	0.429	0.452	0.430
**$NICS_{zz}(1.7)$**	C_5	17.6	-	-	22.8
	C_7	29.4	-	-	34.7

[a] Geometric index; without using a calculated wavefunction.
[b] Current software implementation is not compatible with multireference methods.

Table S6: EDDB electron densities of T_1 azulene in its optimized minimum-energy geometry at U-B3LYP/def2-TZVPP level of theory; $EDDB_G$ – density of globally delocalized electrons in bonds, $EDDB_H$ – density of globally delocalized electrons in bonds of heavy atoms, $EDDB_F$ – density of delocalized bonds in bonds of fragments (local contributions), $EDDB_E$ – density of delocalized electrons in bonds of fragments (local+non-local contributions), $EDDB_P$ – density of delocalized electrons in bonds along a pathway. Rings C_5, C_7 and C_{10} assigned per scheme in Figure S6.

Index	Fragment/path	Electron density	Electron density(α)	Electron density(β)
**$EDDB_G$**	-	8.7	4.6	4.1
**$EDDB_H$**	-	8.0	4.3	3.7
**$EDDB_F$**	C_5	2.9	2.3	0.6
	C_7	4.2	1.5	2.7
**$EDDB_E$**	C_5	4.6	2.8	1.8
	C_7	6.0	2.8	3.2
**$EDDB_P$**	C_5	2.4	2.0	0.4
	C_7	3.2	0.9	2.3
	C_{10}(perimeter)	4.5	2.3	2.2
	C_{10}(transannular)	5.8	3.0	2.8

Table S7: Active space orbital energies and normalized densities of T_1 azulene, calculated at CASSCF(10,10)/def2-TVPP.

Active Space Orbital	Orbital energy (eV)	Normalized density
1	-13.73	1.95
2	-11.81	1.93
3	-9.97	1.89
4	-8.31	1.89
5	-3.96	1.02
6	-1.73	0.99
7	3.20	0.14
8	7.46	0.08
9	9.67	0.06
10	10.29	0.05

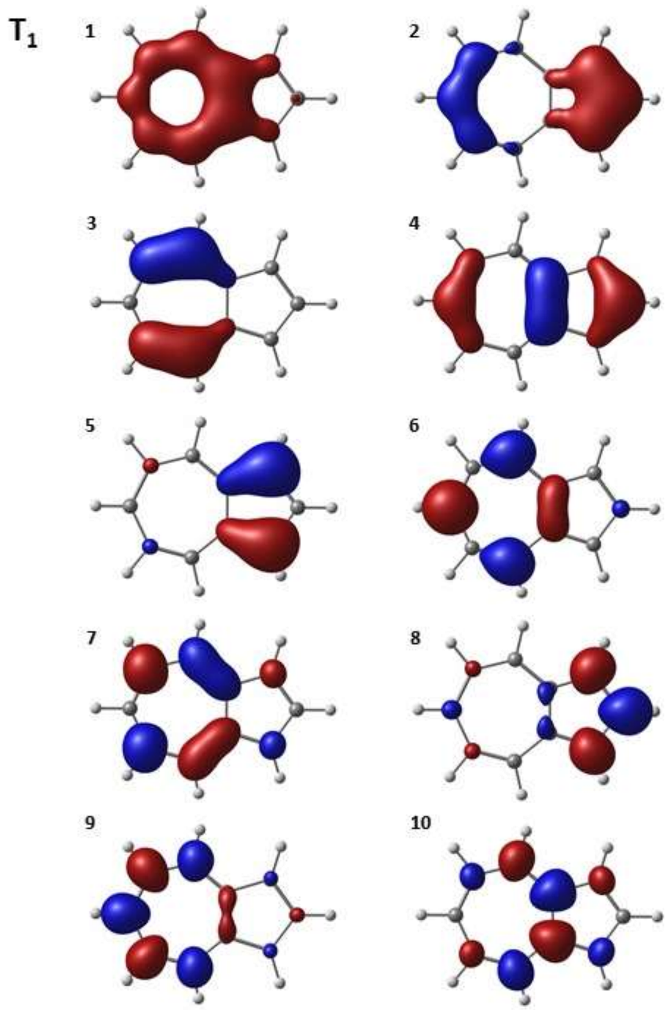

Figure S11: Active MOs of T$_1$ azulene, calculated at CASSCF(10,10)/def2-TVPP.

Table S8: CI vector of the T_1 azulene, calculated at CASSCF(10,10)/def2-TVPP (configuration printed as a string of orbital occupancies, in decreasing order of active MOs).

Configuration	Weight
2222110000	0.788
2212111000	0.021
2122110100	0.015
2221110100	0.011
2202112000	0.009
2122110010	0.006
2221201000	0.006
2221110010	0.005
2121110200	0.005
2211121000	0.004
1212111010	0.004
1222110010	0.004
2212101010	0.004
2112111010	0.004
2212110001	0.004
2220110200	0.004
2121211000	0.004
2211220000	0.004
2221101001	0.003
2220112000	0.003
2022110200	0.003
1222101001	0.003
1221111001	0.003
1221210001	0.003
1222110100	0.003

S5.3 First quintet excited state (Qu_1) azulene

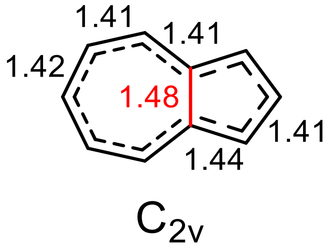

Figure S12: Summary of bond lengths of Qu_1 azulene in its optimized minimum-energy geometry (point group C_{2v}) U-B3LYP/def2-TZVPP.

Table S9: Summary of aromaticity indices for Qu_1 azulene in its optimized minimum-energy geometry, harmonic oscillator model of aromaticity (HOMA), ring-size normalized multicentre bond index (MCI), aromatic fluctuation index (FLU), ring-size normalized Giambagi multicentre index (I_{ring}) and the zz component of the nucleus independent chemical shift 1.7 Å above the centre of the ring ($NICS_{zz}(1.7)$), calculated at UB3LYP/6-311+G** (UKS), and CASSCF(10,10)/def2-TZVPP for MCI and CASSCF(10,10)/6-311+G**for NICS (CAS-SCF). Rings C_5, C_7 and C_{10} assigned per scheme in Figure S6.

Index	Ring	UKS(net)	UKS(α)	UKS(β)	CAS-SCF
**HOMA**	C_5	0.22	[a]	[a]	[a]
	C_7	0.51	[a]	[a]	[a]
	C_{10}	0.73	[a]	[a]	[a]
**$MCI^{1/n}$**	C_5	0.515	0.418	0.472	0.390
	C_7	0.433	0.442	-0.336	0.357
	C_{10}	0.619	0.510	0.609	0.429
**FLU**	C_5	0.024	0.012	0.041	[b]
	C_7	0.015	0.007	0.028	[b]
	C_{10}	0.011	0.005	0.022	[b]
**$I_{ring}^{1/n}$**	C_5	0.418	0.379	0.346	0.386
	C_7	0.390	0.306	0.581	0.366
	C_{10}	0.451	0.339	0.448	0.412
**$NICS_{zz}(1.7)$**	C_5	-19.0	-	-	-15.2
	C_7	-20.3	-	-	-16.5

[a] Geometric index; does not use a calculated wavefunction.
[b] Current software implementation is not compatible with multireference methods.

Table S10: EDDB electron densities of Qu_1 azulene in its optimized minimum-energy geometry at U-B3LYP/def2-TZVPP level of theory; $EDDB_G$ – density of globally delocalized electrons in bonds, $EDDB_H$ –

density of globally delocalized electrons in bonds of heavy atoms, EDDB$_F$ – density of delocalized bonds in bonds of fragments (local contributions), EDDB$_E$ – density of delocalized electrons in bonds of fragments (local+non-local contributions), EDDB$_P$ – density of delocalized electrons in bonds along a pathway. Rings C_5, C_7 and C_{10} assigned per scheme in Figure S6.

Index	Fragment/path	Electron density	Electron density(α)	Electron density(β)
**EDDB$_G$**	-	8.7	5.8	2.9
**EDDB$_H$**	-	8.0	5.5	2.5
**EDDB$_F$**	C_5	2.4	1.9	0.5
	C_7	4.1	3.1	1.1
**EDDB$_E$**	C_5	4.2	2.9	1.3
	C_7	6.1	4.0	2.1
**EDDB$_P$**	C_5	1.7	1.4	0.3
	C_7	2.8	2.1	0.7
	C_{10}(perimeter)	4.9	3.4	1.5
	C_{10}(transannular)	5.1	3.6	1.5

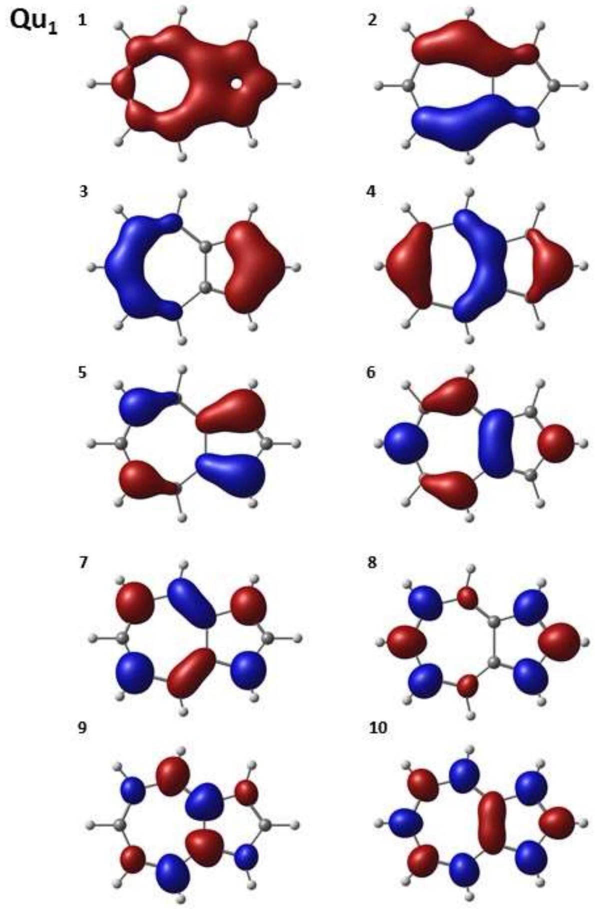

Figure S13: Active MOs of Qu_1 azulene, calculated at CASSCF(10,10)/def2-TVPP.

Table S11: Active space orbital energies and normalized densities of Qu_1 azulene, calculated at CASSCF(10,10)/def2-TVPP.

Active Space Orbital	Orbital energy (eV)	Normalized density
1	-13.41	1.93
2	-9.98	1.88
3	-10.90	1.85
4	-6.05	1.09
5	-4.66	1.07
6	-0.85	0.94
7	0.27	0.94
8	6.79	0.14
9	9.43	0.09
10	8.71	0.08

Table S12: CI vector of the Qu_1 azulene, calculated at CASSCF(10,10)/def2-TVPP (configuration printed as a string of orbital occupancies, in decreasing order of active MOs).

Configuration	Weight
2221111000	0.736
2212101100	0.036
2211210100	0.029
2121210010	0.017
2112211000	0.016
2122101010	0.015
1222101001	0.012
1221210001	0.010
2211111001	0.009
1221111100	0.006
2210121100	0.005
2211012100	0.005
2221100110	0.005
1211111101	0.004
1121111011	0.003
2201111200	0.003
2121120001	0.003
2122110100	0.003
2121012010	0.003

S5.4 First excited singlet (S_1) and second excited singlet (S_2) state azulene

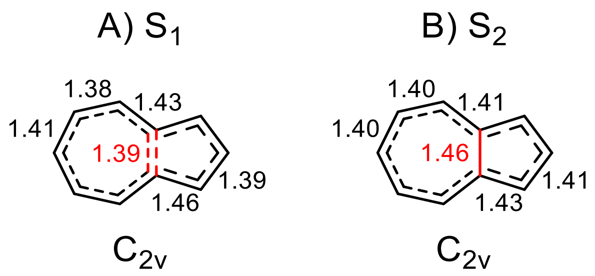

Figure S14: Summary of bond lengths of S_1 and S_2 azulene in its optimized minimum-energy geometries (point group C_{2v}) TD-B3LYP/def2-TZVPP.

Table S13: Summary of aromaticity indices for S_1 and S_2 azulene in its optimized minimum-energy geometry (TD-B3LYP geometry optimization); harmonic oscillator model of aromaticity (HOMA), ring-size normalized multicenter bond index (MCI), ring-size normalized Giambagi multicenter index (I_{ring}) and zz component of the nucleus independent shift 1.7 Å above the centre of the ring ($NICS_{zz}(1.7)$), calculated at CASSCF(10,10)/def2-TZVPP level of theory for MCI and CASSCF(10,10)/6-311+G**for NICS. Rings C_5, C_7 and C_{10} assigned per scheme in Figure S6.

Index	Ring	S_1	S_2
**HOMA**	C_5	0.50	0.54
	C_7	0.83	0.76
	C_{10}	0.63	0.87
**$MCI^{1/n}$**	C_5	0.421	0.425
	C_7	0.484	0.474
	C_{10}	-0.390	0.456
**$I_{ring}^{1/n}$**	C_5	0.443	0.432
	C_7	0.474	0.455
	C_{10}	0.434	0.425
**$NICS_{zz}(1.7)$ CAS**	C_5	16.4	-15.4
	C_7	13.7	-11.3

Table S14: EDDB electron densities of S_1 azulene in its optimized minimum-energy geometry (TD-B3LYP geometry optimization, CASSCF(10,10)/def2-TZVPP single point); $EDDB_G$ – density of globally delocalized electrons in bonds, $EDDB_H$ – density of globally delocalized electrons in bonds of heavy atoms, $EDDB_F$ – density of delocalized bonds in bonds of fragments (local contributions), $EDDB_E$ – density of delocalized electrons in bonds of fragments (local+non-local contributions), $EDDB_P$ – density of delocalized electrons in bonds along a pathway. Rings C_5, C_7 and C_{10} assigned per scheme in Figure S6.

Index	Fragment/path	Electron density
**$EDDB_G$**	-	7.2
**$EDDB_H$**	-	6.7
**$EDDB_F$**	C_5	2.5
	C_7	3.5
**$EDDB_E$**	C_5	3.7
	C_7	4.9
**$EDDB_P$**	C_5	2.0
	C_7	2.5
	C_{10}(perimeter)	3.7
	C_{10}(transannular)	4.6

Table S15: EDDB electron densities of S_2 azulene in its optimized minimum-energy geometry (TD-B3LYP geometry optimization, CASSCF(10,10)/def2-TZVPP single point); $EDDB_G$ – density of globally delocalized electrons in bonds, $EDDB_H$ – density of globally delocalized electrons in bonds of heavy atoms, $EDDB_F$ – density of delocalized bonds in bonds of fragments (local contributions), $EDDB_E$ – density of delocalized electrons in bonds of fragments (local+non-local contributions), $EDDB_P$ – density of delocalized electrons in bonds along a pathway. Rings C_5, C_7 and C_{10} assigned per scheme in Figure S6.

Index	Fragment/path	Electron density
**$EDDB_G$**	-	7.9
**$EDDB_H$**	-	7.4
**$EDDB_F$**	C_5	2.7
	C_7	4.2
**$EDDB_E$**	C_5	3.9
	C_7	5.5
**$EDDB_P$**	C_5	2.3
	C_7	2.9
	C_{10}(perimeter)	4.6
	C_{10}(transannular)	5.2

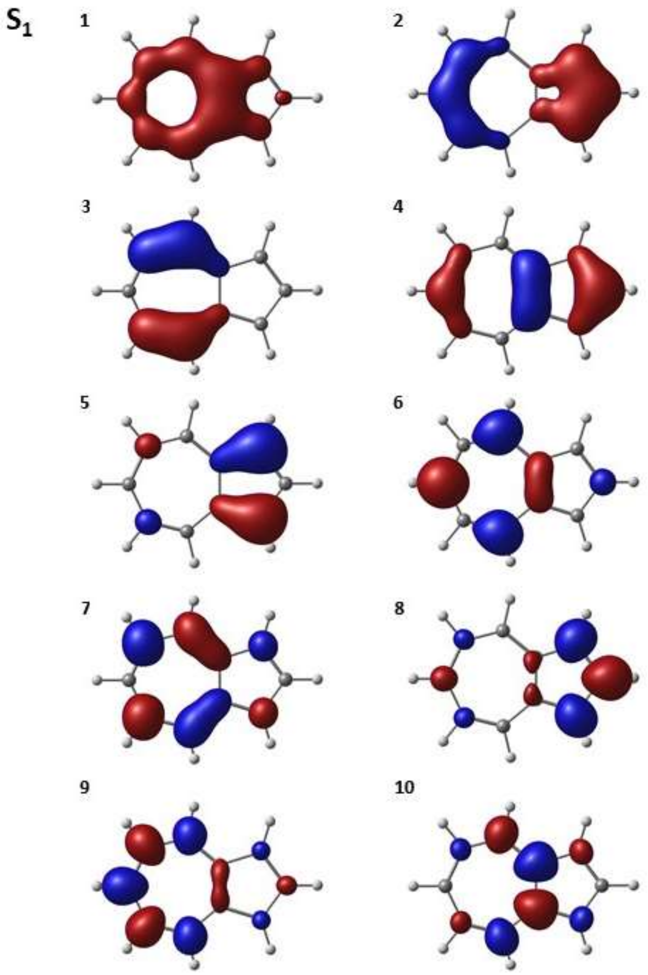

Figure S15: Active MOs of S_1 azulene, calculated at CASSCF(10,10)/def2-TVPP.

Table S16: Active space orbital energies and normalized densities of S_1 azulene, calculated at CASSCF(10,10)/def2-TVPP.

**Active Space Orbital**	**Orbital energy (eV)**	**Normalized density**
1	-13.79	1.95
2	-11.73	1.92
3	-9.94	1.88
4	-8.25	1.87
5	-3.99	1.03
6	-1.65	0.98
7	3.12	0.16
8	7.42	0.09
9	9.66	0.06
10	10.35	0.05

Table S17: CI vector of the S_1 azulene, calculated at CASSCF(10,10)/def2-TVPP (configuration printed as a string of orbital occupancies, in order of active MOs).

Configuration	Weight
2222110000	0.755
2212111000	0.032
2221110100	0.020
2221201000	0.017
2122110010	0.014
2202112000	0.010
2122110100	0.006
1222110100	0.006
2112111010	0.006
2121211000	0.005
2220110200	0.005
2221101001	0.004
2212101010	0.004
2212110001	0.004
2121110200	0.004
2211121000	0.003
1212111010	0.003
1222101001	0.003
1221210001	0.003
1222110010	0.003

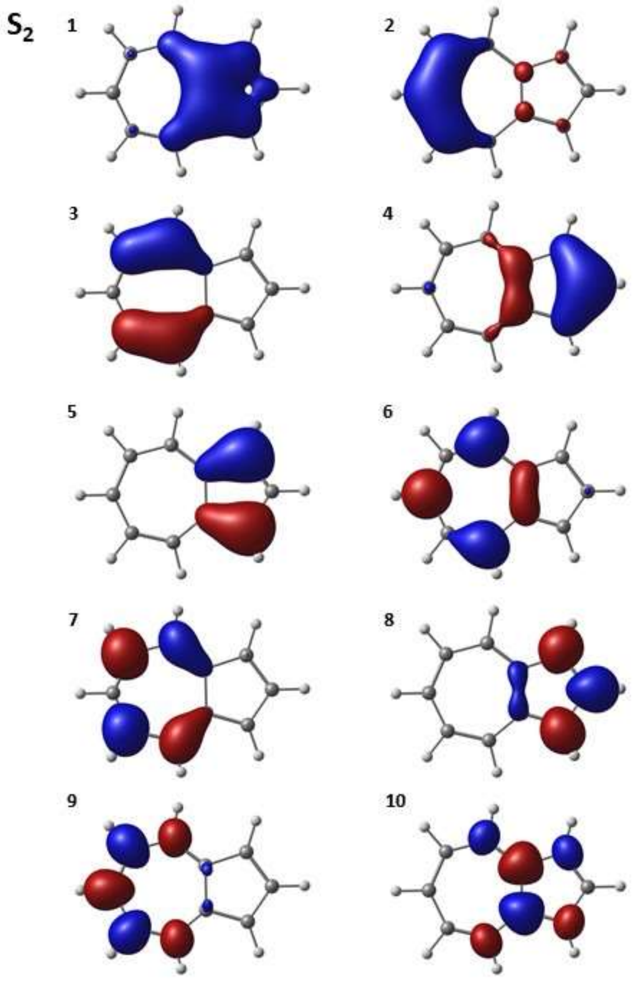

Active space orbital energies and normalized densities of S_2 azulene, calculated at CASSCF(10,10)/def2-TVPP.

Table S18: Active space orbital energies and normalized densities of S_2 azulene, calculated at CASSCF(10,10)/def2-TVPP.

Active MO	Orbital energy (eV)	Normalized density
1	-13.58	1.94
2	-11.19	1.92
3	-9.50	1.83
4	-7.49	1.53
5	-5.32	1.36
6	-0.30	0.61
7	1.15	0.58
8	6.85	0.11
9	9.20	0.07
10	9.58	0.05

Table S19: CI vector of the S_2 azulene, calculated at CASSCF(10,10)/def2-TVPP (configuration printed as a string of orbital occupancies, in decreasing order of active MOs).

Configuration	Weight
2222101000	0.391
2221210000	0.310
2212120000	0.038
2222010100	0.013
2221101100	0.011
2202121000	0.011
2211211000	0.010
2220210100	0.010
2222200000	0.009
2212110010	0.008
1222111000	0.008
2221010200	0.007
2121210010	0.006
2212102000	0.006
2122101010	0.005
2220101200	0.005
2201212000	0.005
1212111001	0.004
2221111000	0.004
2022121000	0.004
2112112000	0.004
1222101100	0.004

S5.5 S_1-S_0 conical intersection

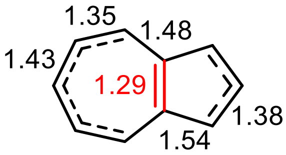

C_s

Figure S16: Summary of bond lengths of azulene in its optimized S_1-S_0 conical intersection geometry (point group C_s) TD-B3LYP/def2-TZVP.

Table S20: Summary of aromaticity indices of the optimized conical intersection geometry of azulene in the S_1 and S_0 electron configurations (TD-B3LYP geometry optimization); harmonic oscillator model of aromaticity (HOMA), ring-size normalized multicenter bond index (MCI), ring-size normalized Giambagi multicenter index (I_{ring}) and zz component of the nucleus independent chemical shift 1.7 Å above the centre of the ring ($NICS_{zz}(1.7)$), calculated at the CASSCF(10,10)/def2-TZVPP level of theory for MCI and CASSCF(10,10)/6-311+G** for NICS (CAS-SCF). Rings C_5, C_7 and C_{10} assigned per scheme in Figure S6.

Index	Ring	S_1	S_0
**HOMA**	C_5	-1.81	
	C_7	-0.28	
	C_{10}	-0.85	
**$MCI^{1/n}$**	C_5	0.396	0.446
	C_7	0.441	0.510
	C_{10}	-0.362	0.548
**$I_{ring}^{1/n}$**	C_5	0.422	0.459
	C_7	0.432	0.479
	C_{10}	0.377	0.497
**$NICS_{zz}(1.7)$ CAS**	C_5	$-1.01^{a}/0.99^{b}$	-
	C_7	^{a}	^{a}

[a] Distal plane (to the C_7 ring).
[b] Proximal plane (to the C_7 ring).
[c] The C_7 ring cannot be aligned in the xy plane due to its distortion (Figure S17).

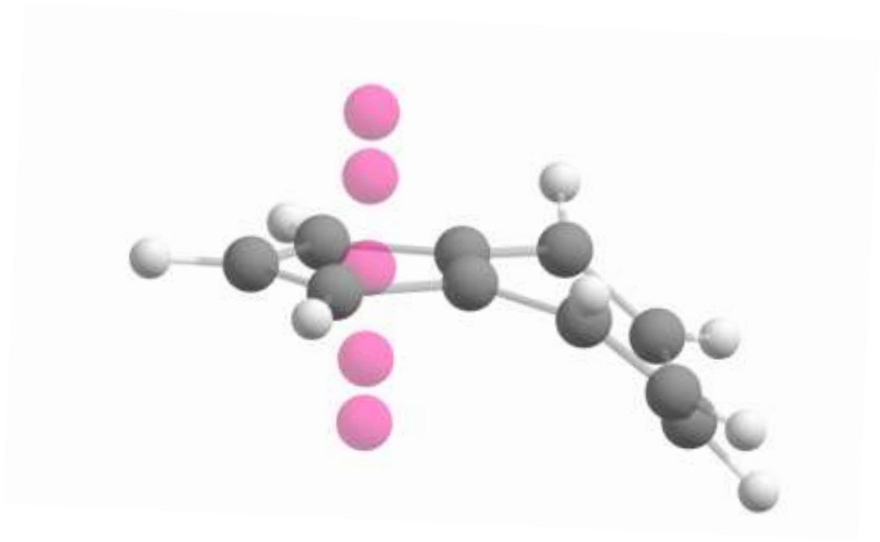

Figure S17: Dummy atoms (pink) used to calculate the $NICS_{zz}(1.7)$ index.

Table S21: EDDB electron densities of the optimized conical intersection geometry of azulene in the S_1 and S_0 electron configuration (TD-B3LYP geometry optimization, CASSCF(10,10)/def2-TZVPP single point); $EDDB_G$ – density of globally delocalized electrons in bonds, $EDDB_H$ – density of globally delocalized electrons in bonds of heavy atoms, $EDDB_F$ – density of delocalized bonds in bonds of fragments (local contributions), $EDDB_E$ – density of delocalized electrons in bonds of fragments (local+non-local contributions), $EDDB_P$ – density of delocalized electrons in bonds along a pathway. Rings C_5, C_7 and C_{10} assigned per scheme in Figure S6.

Index	Fragment/path	S_1 Electron density	S_0 Electron density
**$EDDB_G$**	-	4.9	8.5
**$EDDB_H$**	-	4.4	8.0
**$EDDB_F$**	C_5	2.1	2.8
	C_7	1.9	3.6
**$EDDB_E$**	C_5	2.7	4.5
	C_7	2.8	5.5
**$EDDB_P$**	C_5	1.3	2.5
	C_7	1.1	3.0
	C_{10}(perimeter)	2.3	5.7
	C_{10}(transannular)	2.5	5.0

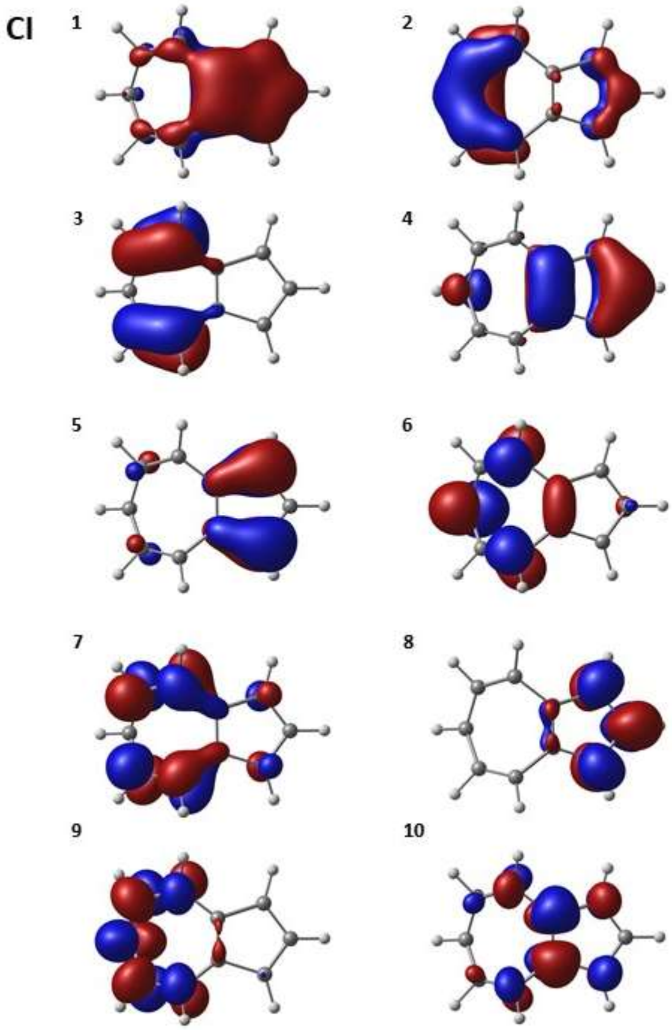

Figure S18: Active MOs of the optimized conical intersection of azulene, calculated at CASSCF(10,10)/def2-TVPP.

Table S22: Active space orbital energies and normalized densities of the optimized conical intersection of azulene, calculated CASSCF(10,10)/def2-TVPP, in the S_1 state.

Active MO	Orbital energy (eV)	Normalized density
1	-13.32	1.95
2	-11.56	1.93
3	-8.70	1.90
4	-9.64	1.89
5	-3.31	1.01
6	-2.56	1.00
7	4.51	0.12
8	7.48	0.08
9	8.68	0.07
10	9.79	0.05

Table S23: CI vector of the optimized conical intersection geometry of azulene, in the S_1 state, calculated at CASSCF(10,10)/def2-TVPP (configuration printed as a string of orbital occupancies, in decreasing order of active MOs).

Configuration	Weight
2222110000	0.782
2212110100	0.026
2221111000	0.026
2122110010	0.020
2220112000	0.011
2121111010	0.010
2202110200	0.007
2222200000	0.006
2222020000	0.006
1222110100	0.006
1212110002	0.004
2122110100	0.004
2112110200	0.004
222110002	0.004
2221101010	0.003
1212210001	0.003
1212111001	0.003

Table S24: Active space orbital energies and normalized densities of the optimized conical intersection of azulene, calculated CASSCF(10,10)/def2-TVPP, in the S_0 state.

Active MO	Orbital energy (eV)	Normalized density
1	-13.68	1.95
2	-11.70	1.93
3	-9.78	1.91
4	-6.08	1.84
5	-7.57	1.76
6	-0.33	0.27
7	3.47	0.16
8	7.80	0.07
9	8.64	0.06
10	9.62	0.06

Table S25: CI vector of the optimized conical intersection geometry of azulene, in the S_0 state, calculated at the CASSCF(10,10)/def2-TVPP (configuration printed as a string of orbital occupancies, in decreasing order of active MOs).

Configuration	Weight
2222200000	0.722
2222020000	0.060
2221111000	0.036
2220202000	0.017
2122110100	0.009
2212110001	0.008
2211201001	0.008
2121201100	0.007
1222110010	0.007
2122110010	0.005
2202220000	0.004
2212111000	0.004
2112201010	0.004
1221201010	0.004
2220200200	0.004
2112211000	0.003
1212210001	0.003
2202202000	0.003

S6 Computational results: Functional dependency of calculated aromaticity indices

Table S26: Summary of the aromaticity indices of S_0 azulene at its optimized minimum-energy geometry calculated using BLYP, B3LYP and CAM-B3LYP exchange correlation functionals and 6-311+G**for the basis set, harmonic oscillator model of aromaticity (HOMA), ring-size normalized multicenter bond index (MCI), aromatic fluctuation index (FLU), ring-size normalized Giambagi multicenter index (I_{ring}) and zz component of the nucleus independent chemical shift 1.7 Å above the centre of the ring ($NICS_{zz}(1.7)$). Rings C_5, C_7 and C_{10} assigned as per the scheme in Figure S6.

Index	Ring	BLYP	B3LYP	CAM-B3LYP
**HOMA**[a]	C_5	0.17	0.37	0.47
	C_7	0.45	0.57	0.63
	C_{10}	0.93	0.98	0.99
**$MCI^{1/n}$**	C_5	0.464	0.460	0.458
	C_7	0.556	0.557	0.559
	C_{10}	0.649	0.647	0.646
**FLU_m**	C_5	0.075	0.073	0.072
	C_7	0.075	0.073	0.072
	C_{10}	0.004	0.003	0.003
**$NICS_{zz}(1.7)$**	C_5	-29.2	-30.1	-30.6
	C_7	-18.4	-19.1	-19.3

Table S27: Summary of the aromaticity indices of T_1 azulene at its optimized minimum-energy geometry calculated using unrestricted BLYP, B3LYP and CAM-B3LYP exchange correlation functionals and 6-311+G** for the basis set, harmonic oscillator model of aromaticity (HOMA), ring-size normalized multicenter bond index (MCI), aromatic fluctuation index (FLU), ring-size normalized Giambagi multicenter index (I_{ring}) and zz component of the nucleus independent chemical shift 1.7 Å above the centre of the ring ($NICS_{zz}(1.7)$). Rings C_5, C_7 and C_{10} assigned as per the scheme in Figure S6.

Index	Ring	U-BLYP	U-B3LYP	U-CAM-B3LYP
**HOMA**	C_5	0.41	0.55	0.55
	C_7	0.77	0.85	0.82
	C_{10}	0.59	0.67	0.65
**$MCI^{1/n}$**	C_5	0.506	0.503	0.496
	C_7	0.574	0.579	0.577
	C_{10}	-0.599	-0.584	-0.565
**FLU_m**	C_5	0.033	0.036	0.044
	C_7	0.016	0.018	0.023
	C_{10}	0.033	0.036	0.044
**$NICS_{zz}(1.7)$**	C_5	21.6	17.6	12.7
	C_7	34.0	29.4	22.3

Table S28: Summary of the aromaticity indices of Qu_1 azulene at its optimized minimum-energy geometry, calculated using BLYP, B3LYP and CAM-B3LYP exchange correlation functionals and 6-311+G** for the basis set, harmonic oscillator model of aromaticity (HOMA), ring-size normalized multicenter bond index (MCI), aromatic fluctuation index (FLU), ring-size normalized Giambagi multicenter index (I_{ring}) and the zz component of the nucleus independent chemical shift 1.7 Å above the centre of the ring ($NICS_{zz}(1.7)$). Rings C_5, C_7 and C_{10} assigned as per the scheme in Figure S6.

Index	Ring	U-BLYP	U-B3LYP	U-CAM-B3LYP
**HOMA**	C_5	-0.06	0.22	0.32
	C_7	0.28	0.51	0.52
	C_{10}	0.56	0.73	0.67
**$MCI^{1/n}$**	C_5	0.513	0.515	0.507
	C_7	0.441	0.433	0.443
	C_{10}	0.623	0.619	0.594
**FLU_m**	C_5	0.061	0.058	0.056
	C_7	0.061	0.058	0.056
	C_{10}	0.022	0.023	0.036
**$NICS_{zz}(1.7)$**	C_5	-18.6	-19.0	-16.6
	C_7	-19.9	-20.3	-17.7

S7 Computational results: NICS-xy and NICS-z scan

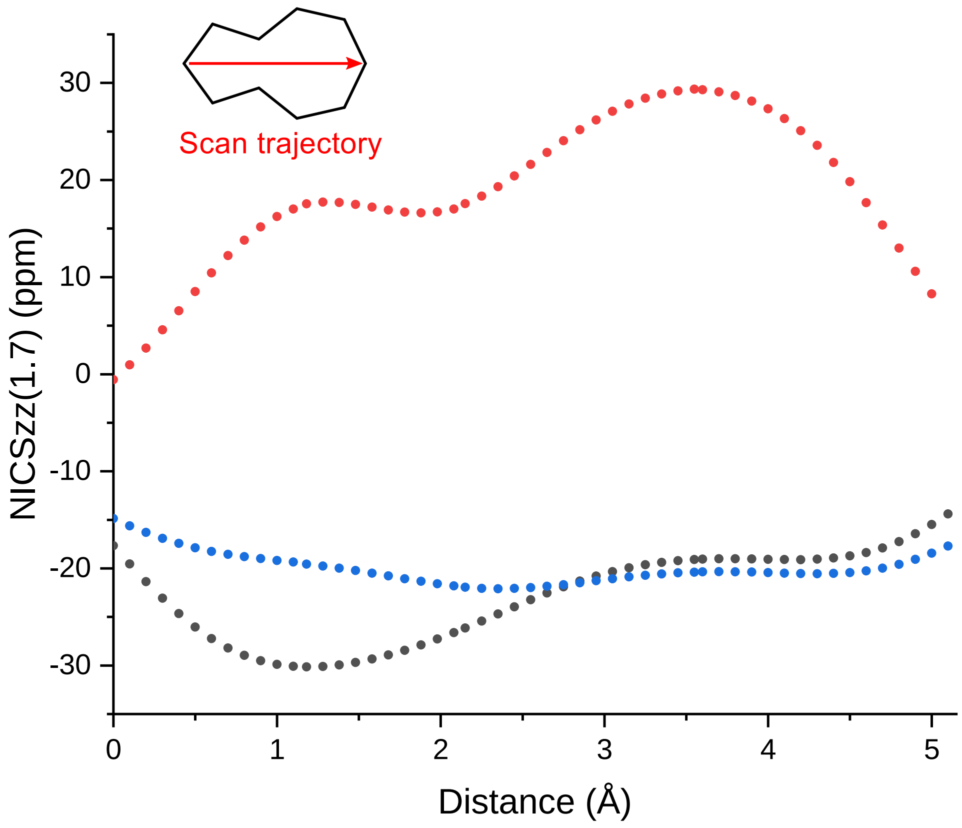

Figure S19: NICS-xy scan of azulene in the minimum-energy geometry of the S_0 (grey), T_1 (red) and Qu_1 (blue) states, calculated at the B3LYP/6-311+G** level of theory.

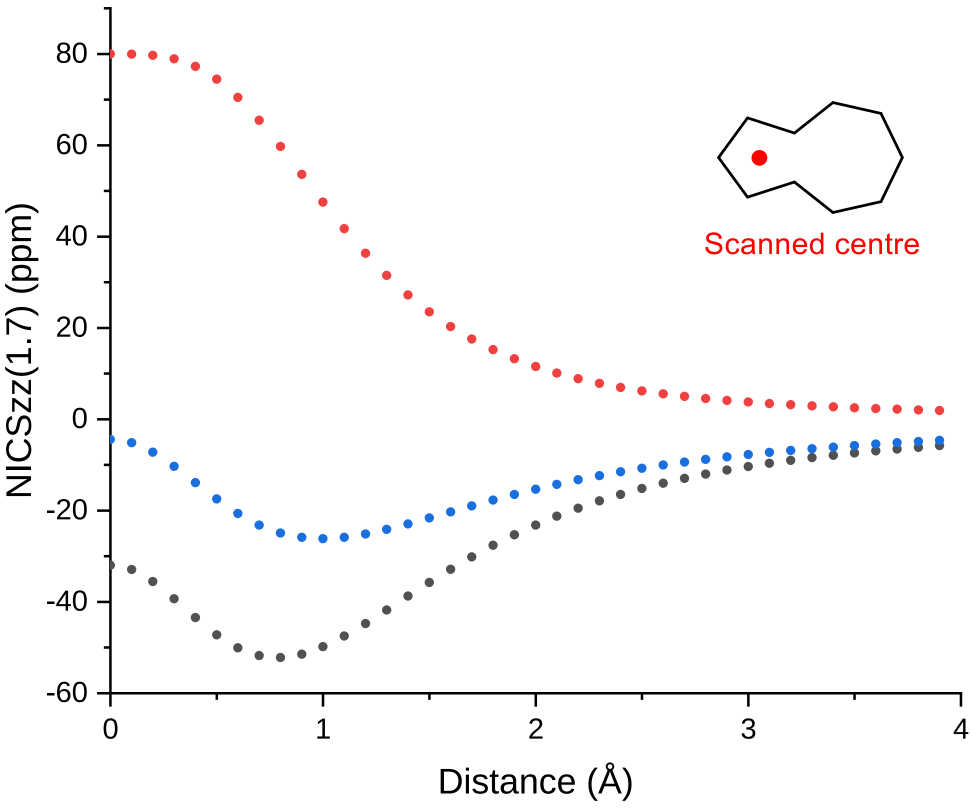

Figure S20: NICS-z scan of the azulene C5 ring in the minimum-energy geometry of the S_0 (grey), T_1 (red) and Qu_1 (blue) states, at the B3LYP/6-311+G** level of theory.

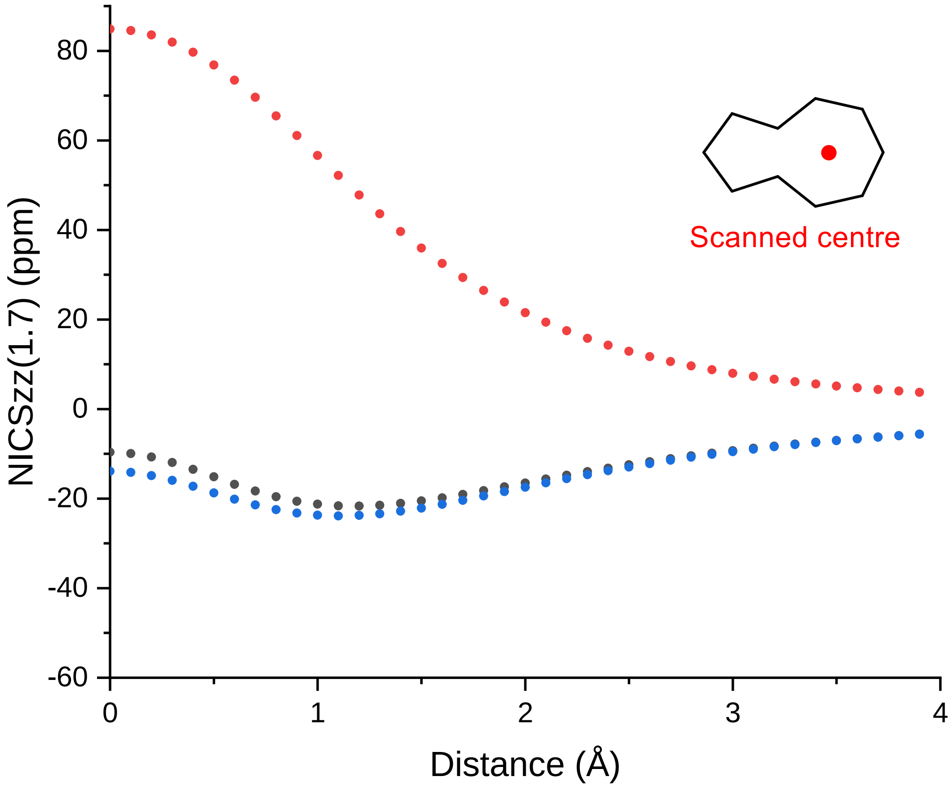

Figure S21: NICS-z scan of the azulene C7 ring in the minimum-energy geometry of the S_0 (grey), T_1 (red) and Qu_1 (blue) states, at the B3LYP/6-311+G** level of theory.

S8 Computational results: Dipole moment of azulene in S_0, T_1 and Qu_1 states

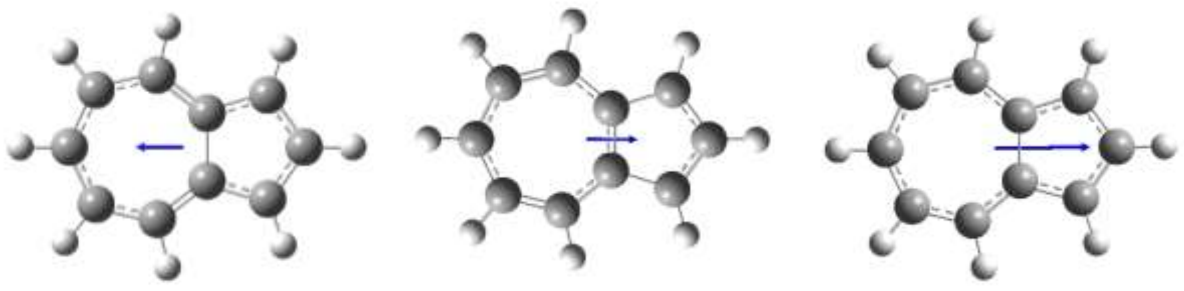

S_0 ***T_1*** ***Qu_1***

Figure S22: Dipole moment vector of azulene in S_0, T_1 and Qu_1 states, superimposed against their optimized minimum energy geometries (and state), calculated at the (U)B3LYP/def2-TZVPP level of theory.

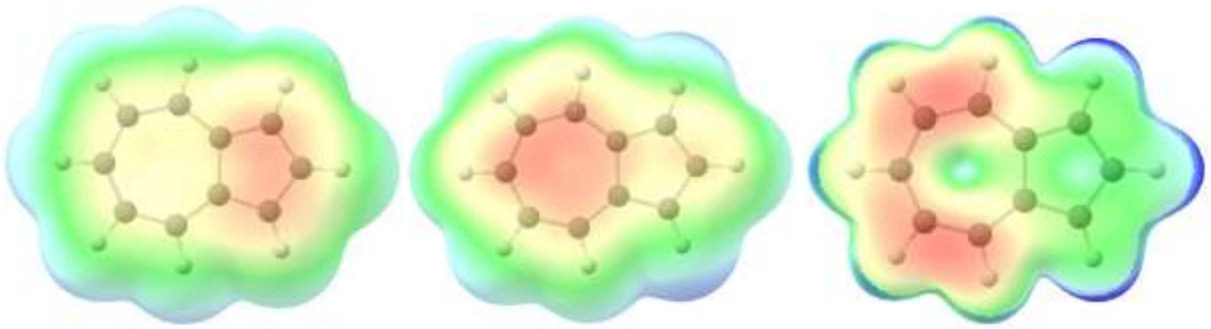

S_0 ***T_1*** ***Qu_1***

Figure S23: Electron-density surface coloured by electrostatic potential of S_0, T_1 and Qu_1 azulene, calculated at the U-B3LYP/def2-TZVPP level of theory (RGB color scheme, red = -0.03 a.u., green = 0.00 a.u., blue = 0.03 a.u.).

Table S29: Components of the dipole moment vectors (D_x, D_y, D_z) of optimized minimum-energy geometries of azulene in S_0, T_1 and Qu_1 states calculated at the (U)B3LYP/def2-TZVPP level of theory, sorted by starting geometry.

**Geometry/state**	D_x	D_y	D_z
S_0	1.03	0.00	0.00
T_1	-0.56	0.00	0.00
Qu_1	-1.94	0.00	0.00

S9 Computational results: Isomerization stabilization energy (ISE) of azulene in the S_0 and T_1 states

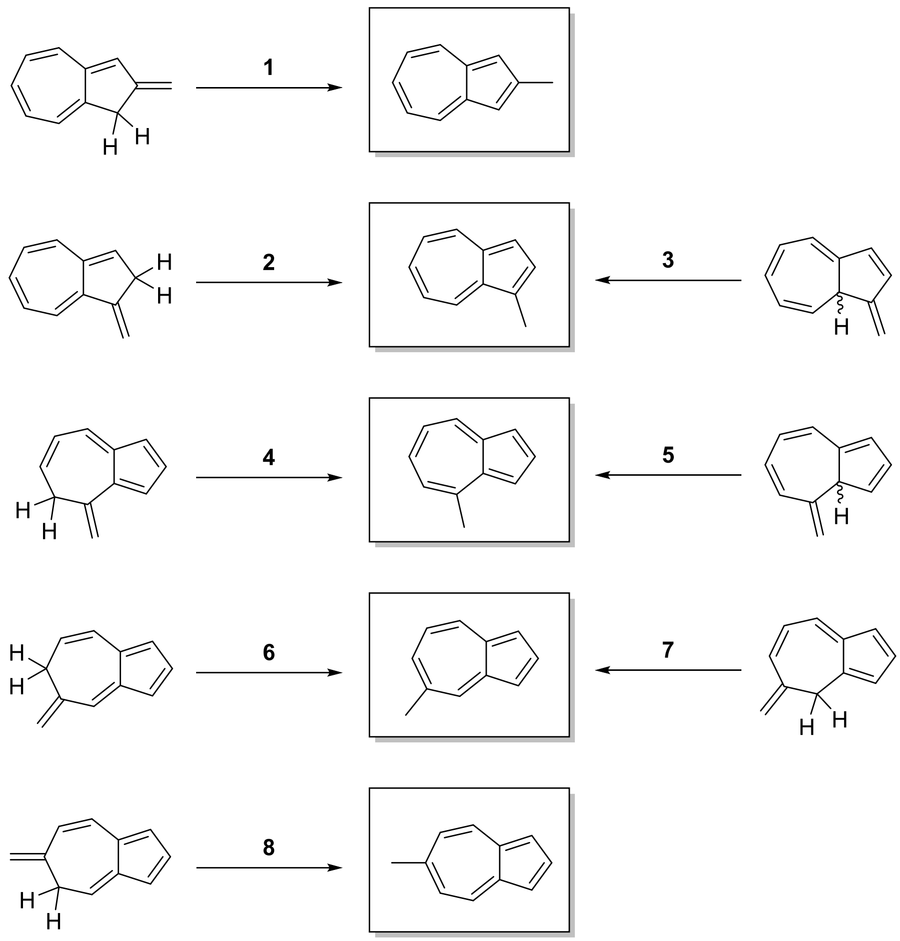

Figure S24: Isomerization reactions applied to the calculation to evaluate S_0 and T_1 ISE.

Table S30: Calculated ISE of S_0 (ISE(S_0)) and T_1 (ISE(T_1)) azulene, following the energies of reactant and products of reactions in Figure S24. Isomers were optimized at (U)B3LYP/def2-TZVPP level of theory; single-point energies were calculated at (U)B3LYP/def2-TZVPP (*RKS*/*UKS*) and (U)-DLPNO-CCSD(T)/def2-TZVPP (*DLPNO-CCSD(T)*/*U-DLPNO-CCSD(T)*) level of theory. The values are colour-coded by magnitude in a three-colour scheme; red = negative, white = 0, blue = positive (shading normalized by minimum and maximum values).

**Reaction**	**ISE(S_0)**		**ISE(T_1)**	
	RKS* *(kcal.mol-1)	***DLPNO-CCSD(T)* *(kcal.mol-1)***	***UKS* *(kcal.mol-1)***	***U-DLPNO-CCSD(T)* *(kcal.mol-1)***
**1**	-17.1	-14.2	0.9	1.4
**2**	-20.5	-16.2	-2.4	-1.4
**3**	-20.9	-15.5	-15.9	-13.8
**4**	-24.0	-18.4	-6.2	-2.9
**5**	-24.6	-19.8	-10.5	-7.6
**6**	-21.9	-16.3	-8.9	-5.8
**7**	-23.3	-17.6	-10.9	-7.5
**8**	-23.3	-17.7	-8.2	-6.7

S10 Experimental results: Absorption and emission spectrum of azulene

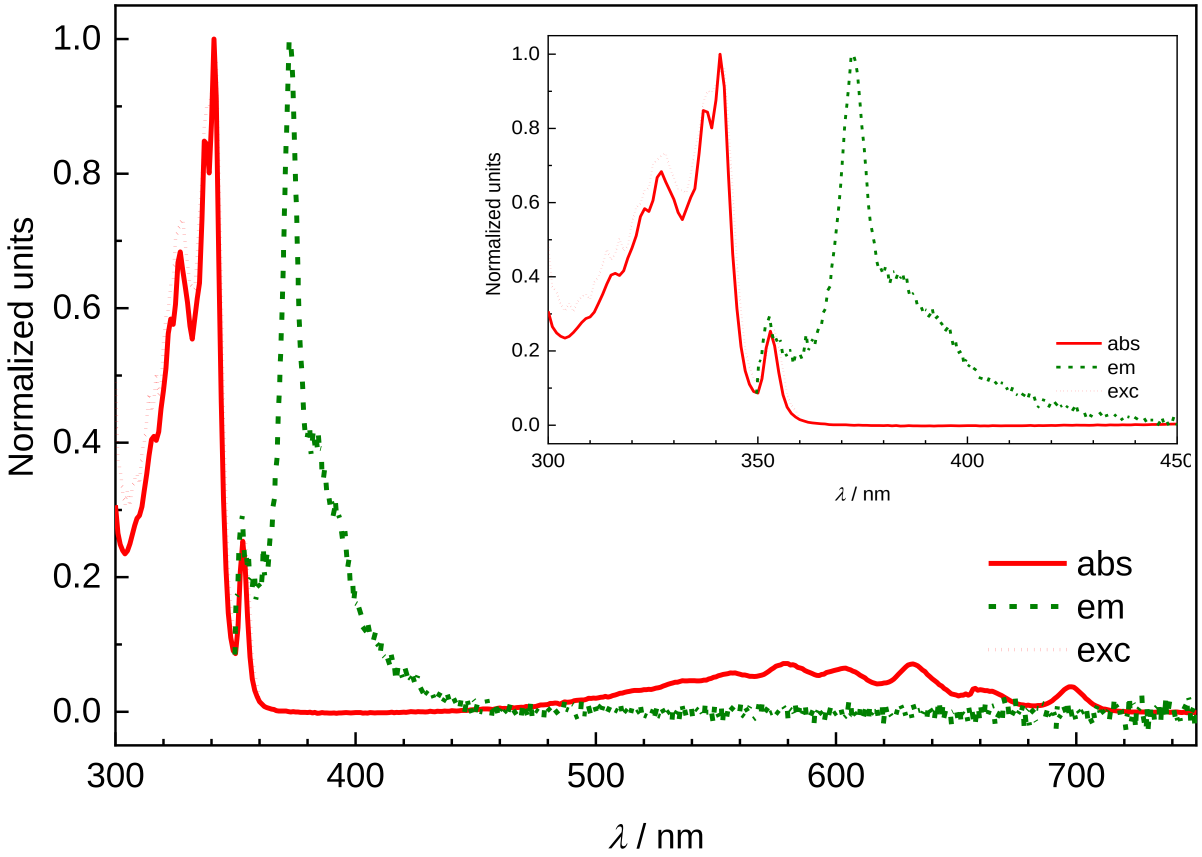

Figure S25: UV-Vis absorption, emission and excitation spectra of azulene in cyclohexane.

S11 Experimental results: Time-resolved spectroscopy of azulene

Laser: Excitation to S_2 state: pump at 350 nm, E = 600 nJ, average of 4 scans, no sample change after measurement, concentration of the sample: ~4.4×10^{-3} M (A_{350} 0.6-0.9 over 2mm, side of sharp abs. peak).

Excitation to S_1 state: pump at 700 or 660 nm, E = 2 uJ**,** average of 5 scans, no sample change after measurement, concentration of the sample: ~8.8×10^{-3} M (A_{350} 0.3 over 2mm).

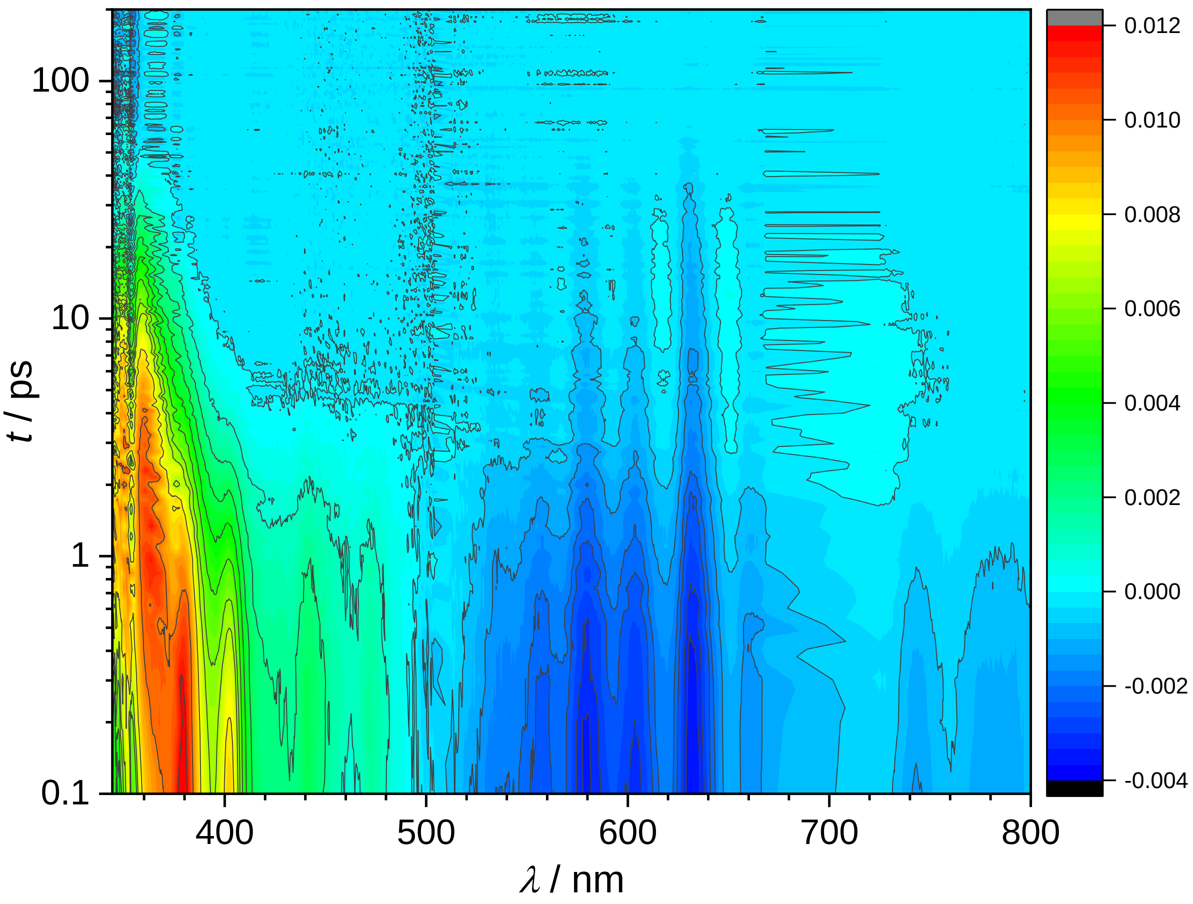

Figure S26: The color map representation of sub-picosecond TAS (transient absorption spectroscopy, of S_1 azulene.

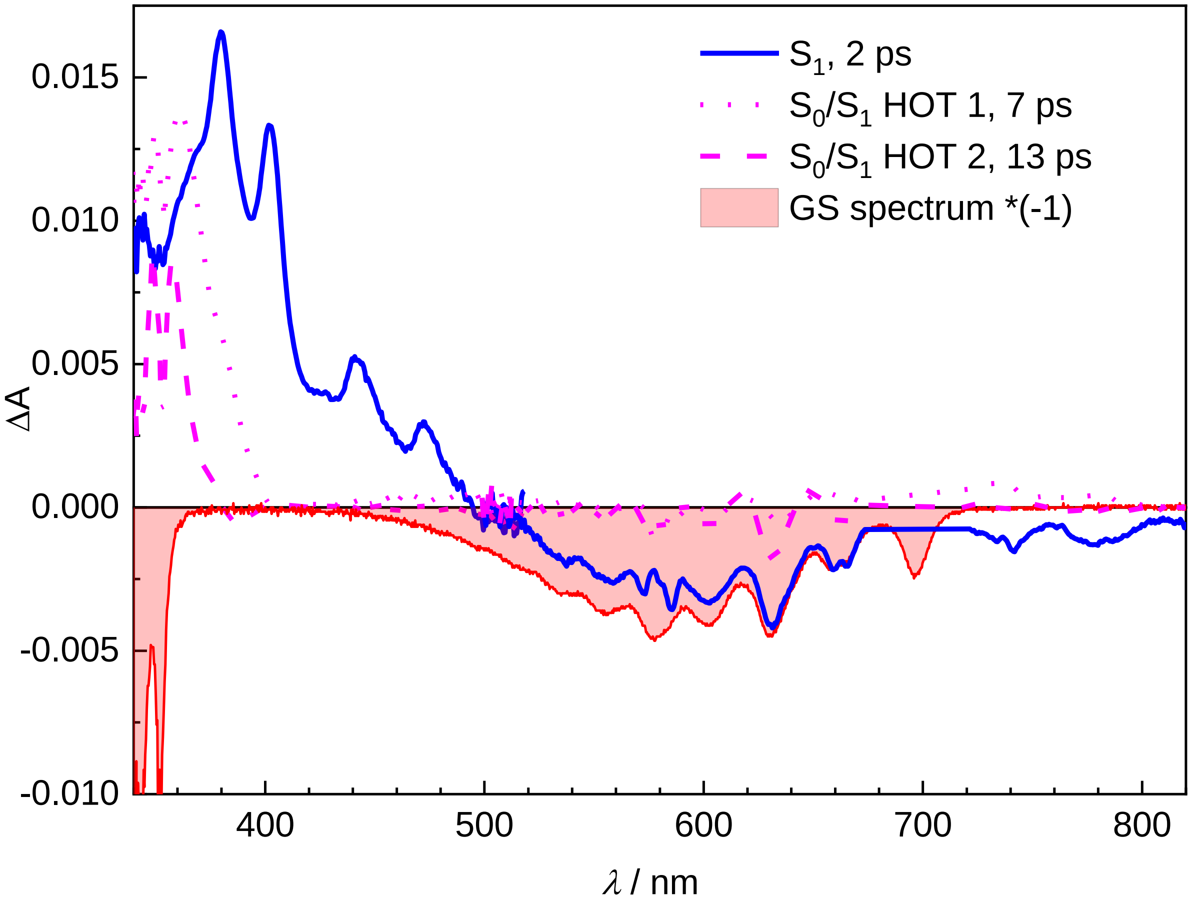

Figure S27: Species associated spectra (SAS) obtained from global analysis of model $S_1 \rightarrow S_0/S_1$ HOT 1 $\rightarrow$ S_0/S_1 HOT 2; obtained from TAS of S_1 azulene.

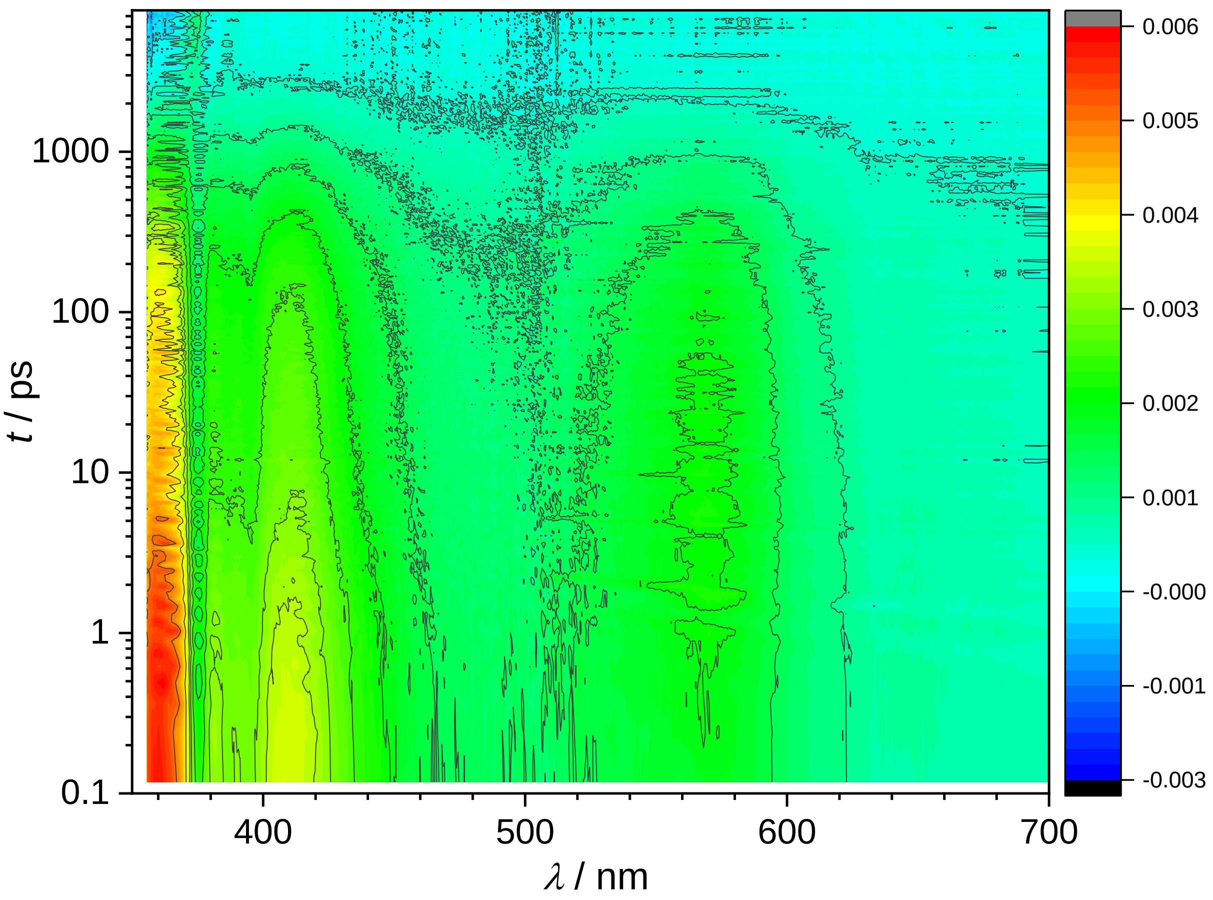

Figure S28: The color map representation of sub-picosecond TAS (transient absorption spectroscopy, of S_2 azulene.

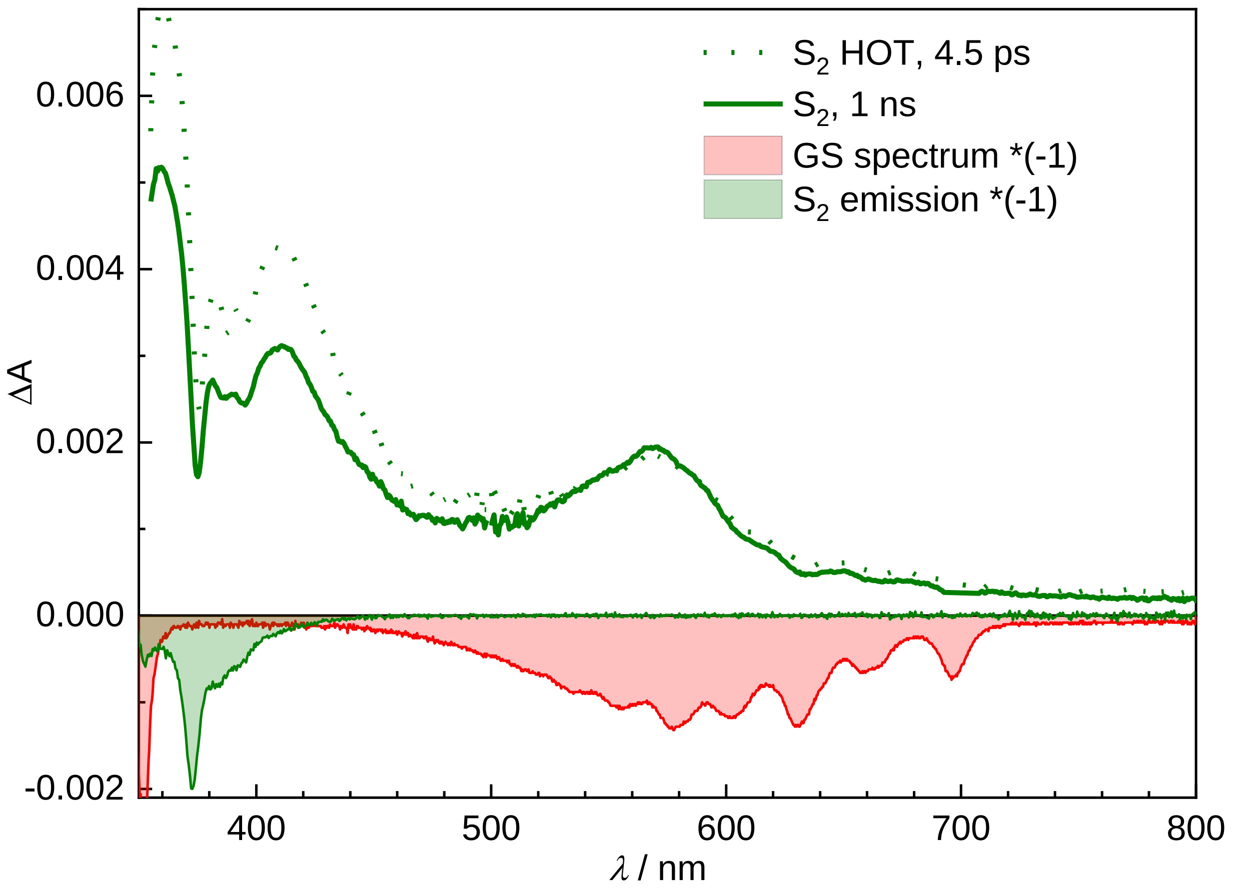

Figure S29: Species associated spectra (SAS) obtained from global analysis of model S_2(HOT) → S_2; obtained from TAS of S_2 azulene.

S12 Extended discussion: Electronic structure of T_1 azulene compared to that of cyclopentadienyl and cycloheptatrienyl radicals

In this study, we have proposed that T_1 azulene adopts the electronic structure of the cyclopentadienyl (C_5) and cycloheptatrienyl (C_7) radical to partly alleviate its aromaticity. We tested this hypothesis by analysing the EDDB of the three species and comparing the $EDDB_F$ (local electron delocalized density in bonds) values of the C_5 and C_7 fragments of T_1 azulene to the $EDDB_H$ (global electron delocalized density in bonds) values of the corresponding radicals. We found striking similarities in the net delocalized electron density and in their spin-separated values (Figure S30). However, the C_5 fragment of the T_1 azulene and the corresponding radical do exhibit geometric discrepancies and, moreover, differences in the plotted $EDDB_F$ and $EDDB_H$ densities, respectively.

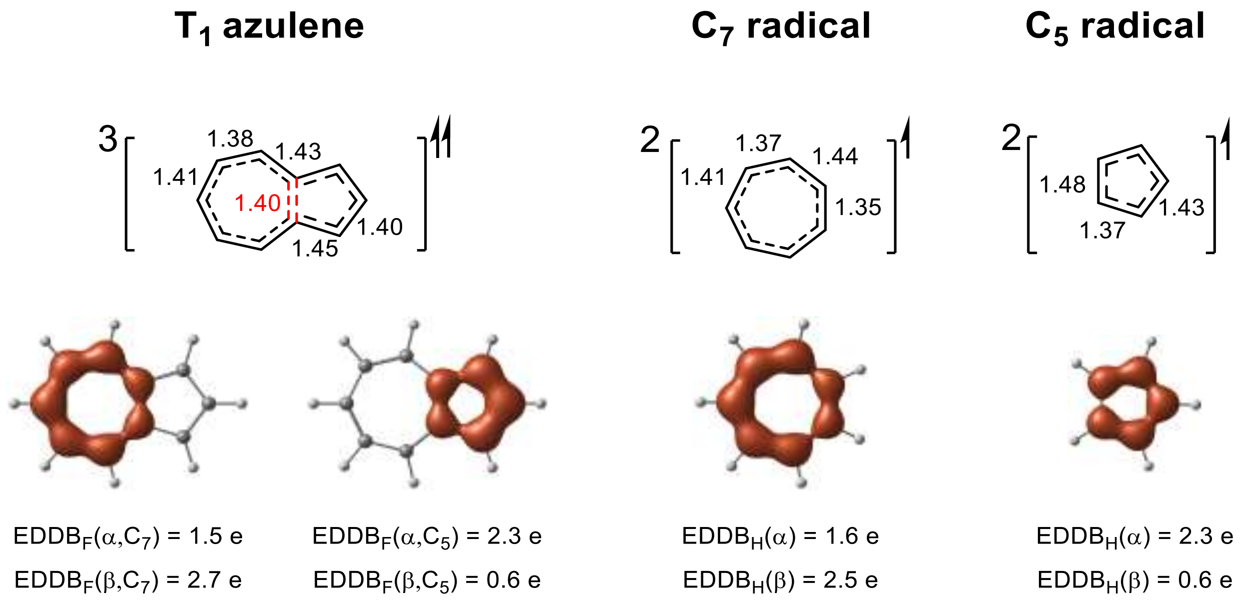

Figure S30: (Left) $EDDB_F$ surface plots of the C_5 and C_7 fragments of T_1 azulene and $EDDB_H$ plots of the (centre) cycloheptatrienyl (C_7) and (right) cyclopentadienyl (C_5) radical, calculated at the U-B3LYP/def2-TZVPP level of theory.

S13 Extended discussion: Azulene compared to naphthalene and homoazulene

S13.1 Homoazulene

Homoazulene,[30] the homoannulene analogue of azulene, provides further insights into the electronic structure of azulene. Owing to the absence of a conjugated transannular bond, unlike azulene, homoazulene cannot exhibit transannular conjugation. Thus, homoazulene serves as an ideal control compound in determining whether resonance structures involving the transannular bond of azulene are also required to explain the dipole moment of azulene or the perimeter delocalization suffices to do so.

Indeed, we found that not only S_0 homoazulene has a significant dipole moment, analogous to that of azulene but homoazulene also undergoes analogous polarity reversal in T_1 and Qu_1 states (Figure S31, Figure S32). Accordingly, the previously discussed ground state resonance structures of azulene, which invoke transannular delocalization (Figure S5), are not required to rationalize its dipole moment.

In the T_1 and Qu_1 states, however, homoazulene adopts a non-conjugated electronic structure (Figure S33). The less aromatic electron structure is most likely reflected in the S_1 energy of homoazulene, which is known to be higher than that of azulene (S_1 absorption maximum at 470 nm).[30] The contrast between the $EDDB_H$ plots of azulene and homoazulene highlights the ability of azulene to adopt the electronic structure of cyclic radicals due to the adaptability of its transannular bond.

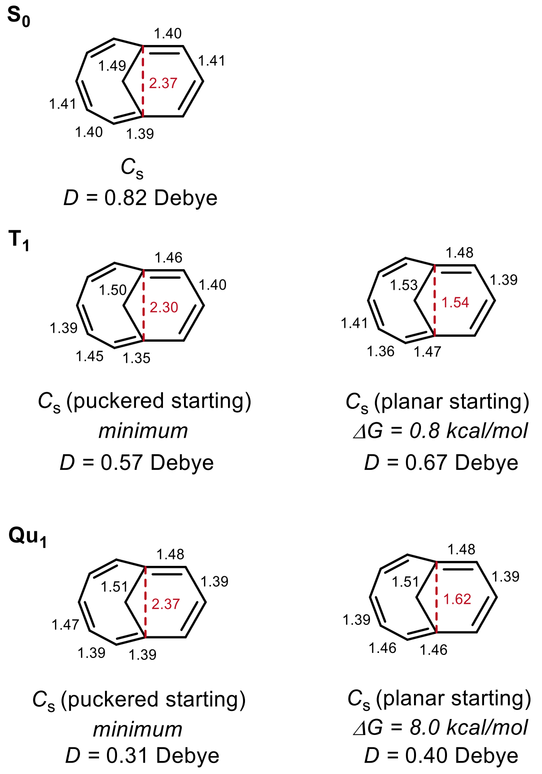

Figure S31: Summary of the key properties of optimized minimum-energy geometries of homoazulene in S_0, T_1 and Qu_1 states, calculated at the (U)B3LYP/def2-TZVPP level of theory; their bond lengths, relative Gibbs energies and dipole moment magnitudes, sorted by starting geometry.

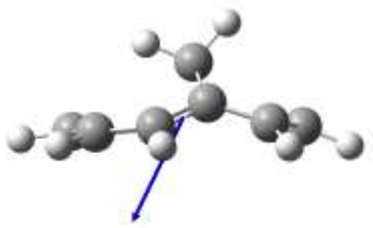

S_0 (single conformer)

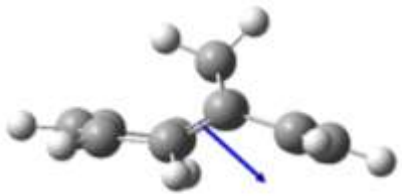

T_1 (puckered starting geom.)

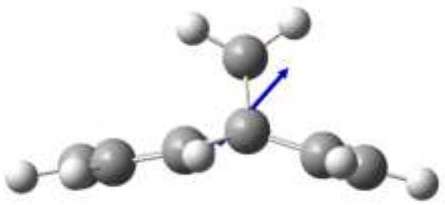

T_1 (planar starting geom.)

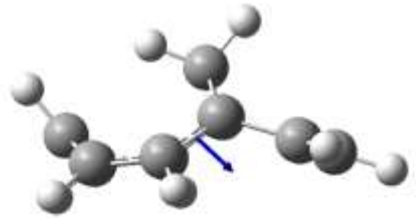

Qu_1 (puckered starting geom.)

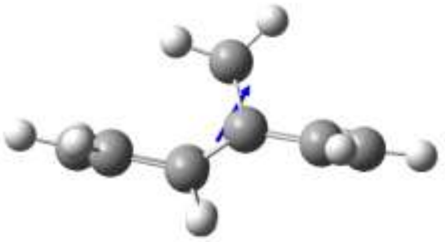

Qu_1 (planar starting geom.)

Figure S32: Dipole moment vectors of optimized minimum-energy geometries of homoazulene in S_0, T_1 and Qu_1 states calculated at the (U)B3LYP/def2-TZVPP level of theory, sorted by starting geometry (vector scaled 3x).

Table S31: Components of the dipole moment vectors (D_x, D_y, D_z) of optimized minimum-energy geometries of homoazulene in S_0, T_1 and Qu_1 states calculated at the (U)B3LYP/def2-TZVPP level of theory, sorted by starting geometry.

**Geometry**	D_x	D_y	D_z
S_0	0.40	0.00	0.71
T_1 (puckered guess)	-0.43	0.01	0.39
T_1 (planar guess)	-0.48	0.00	-0.47
Qu_1 (puckered guess)	-0.21	0.00	0.23
Qu_1 (planar guess)	-0.22	0.00	-0.33

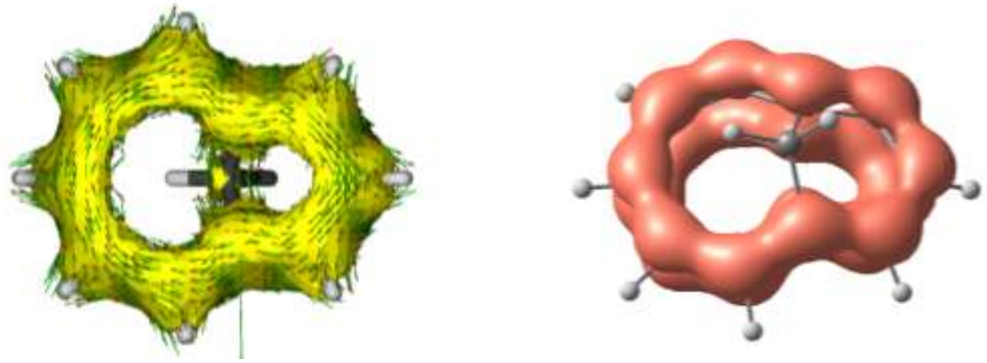

S_0 (single conformer)

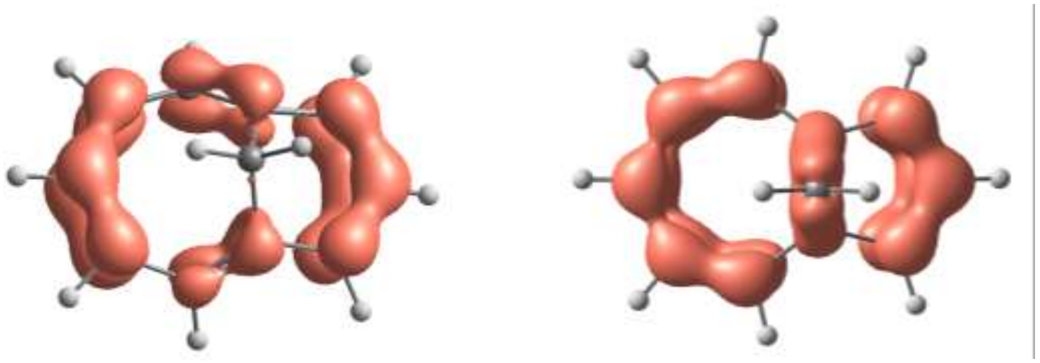

T_1 (puckered starting geom.) ***T_1 (planar starting geom.)***

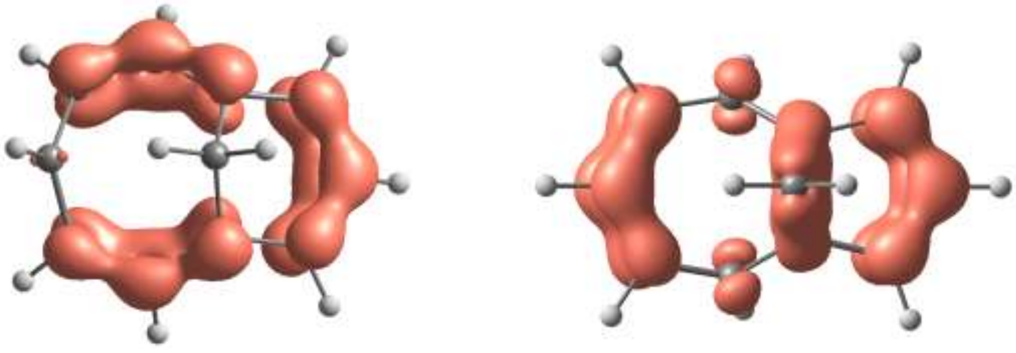

Qu_1 (puckered starting geom.) ***Qu_1 (planar starting geom.)***

Figure S33: $EDDB_H$ plots of optimized minimum-energy geometries of homoazulene in S_0, T_1 and Qu_1 states calculated at the (U)B3LYP/def2-TZVPP level of theory, sorted by starting geometry, and ACID plot of S_0 homoazulene calculated at the B3LYP/6-311+G** level of theory.

S13.2 Naphtalene

Naphtalene is the archetypal example of claromaticity [31]; the electronic structure of closed, 6π-aromatic circuits favored by polycyclic aromatic hydrocarbons.[32-33] Therefore, we used S_0 naphthalene as a control compound to cross-validate our evaluation of the absence of S_0 azulene's transannular delocalization.

Herein, we subtract the $EDDB_P$ densities of naphtalene's "transannular" circuit from its perimeter circuit (Figure S34A). As expected, unlike S_0 azulene, the transannular bond of naphthalene contributes to the global delocalization significantly, by ΔC_{10} = 0.57 electrons, as per the $EDDB_P$ index (Figure S34B). Thus, the ΔC_{10} = 0.02 electrons of S_0 azulene has a negligible contribution to its net delocalized electron density.

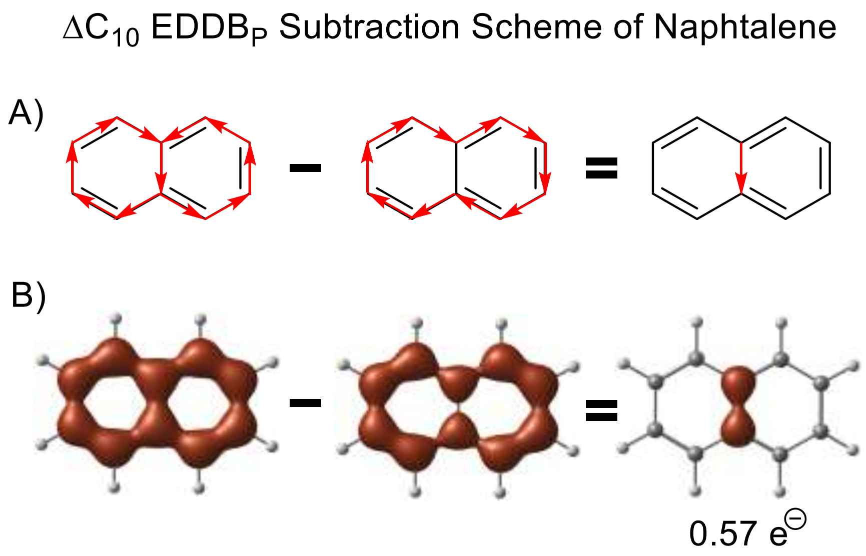

Figure S34: A) Subtraction scheme used to calculate the ΔC_{10} of naphthalene. B) The corresponding $EDDB_P$ plots of optimized S_0 naphtalene calculated at the B3LYP/def2-TZVPP level of theory.

S14 Full-resolution π-ACID plots

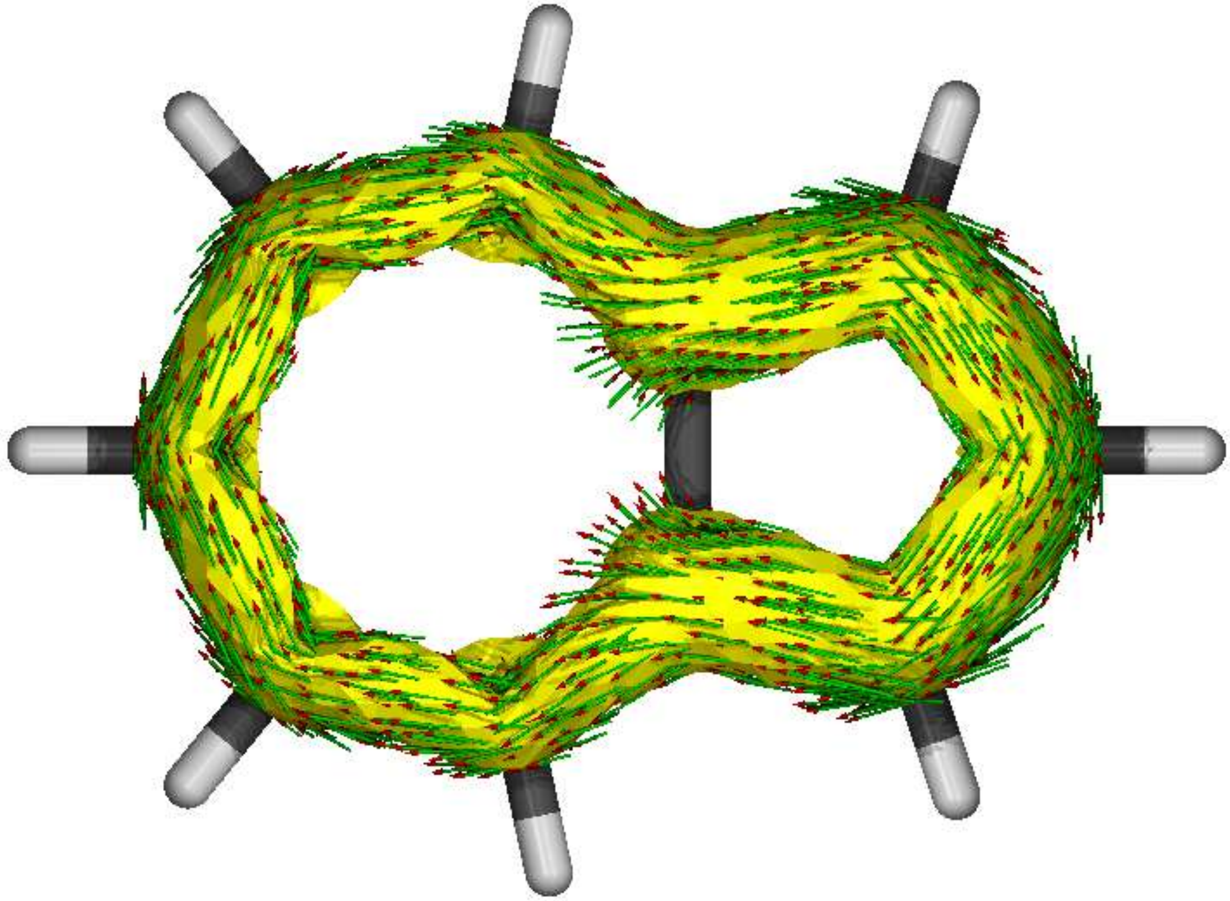

Figure S35: Ground state π-ACID plot of azulene, calculated at B3LYP/6-311+G** level of theory.

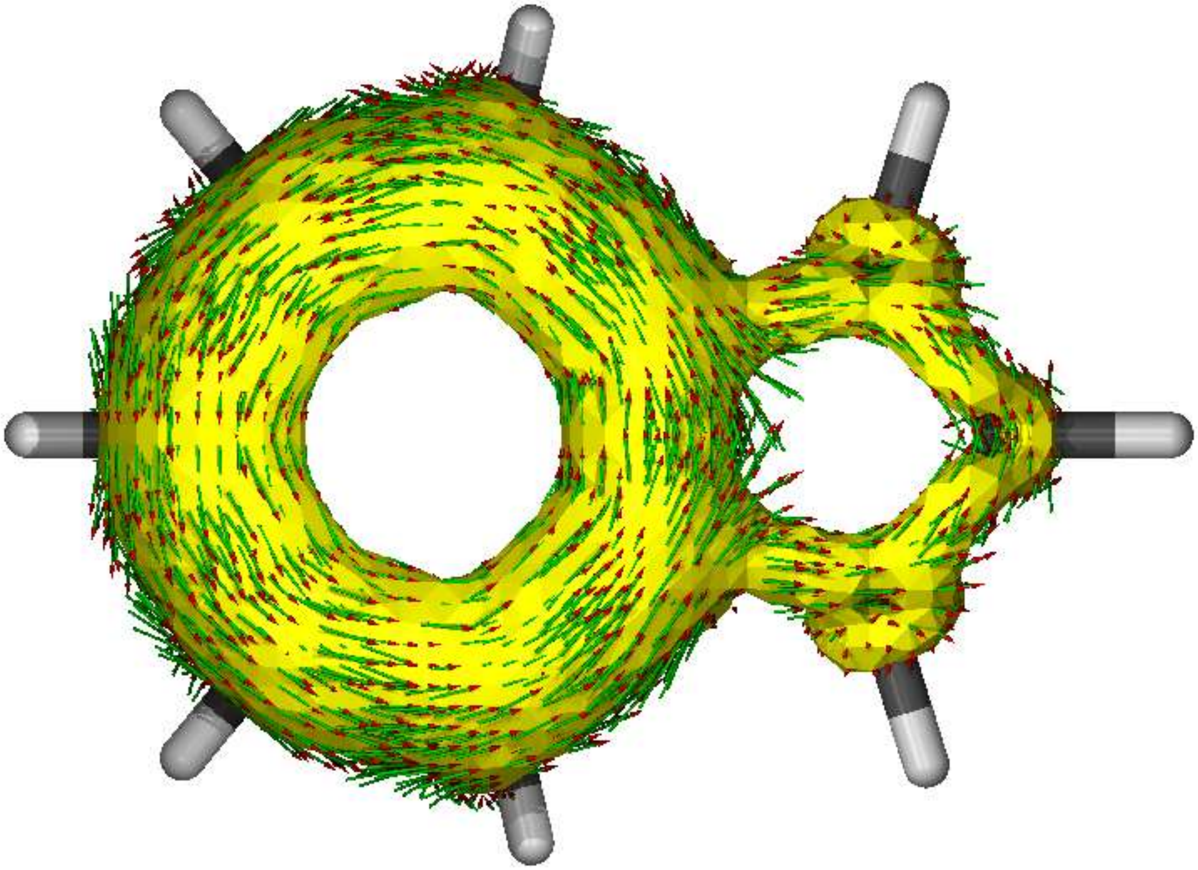

Figure S36: T_1 π-ACID plot of azulene, calculated at B3LYP/6-311+G** level of theory.

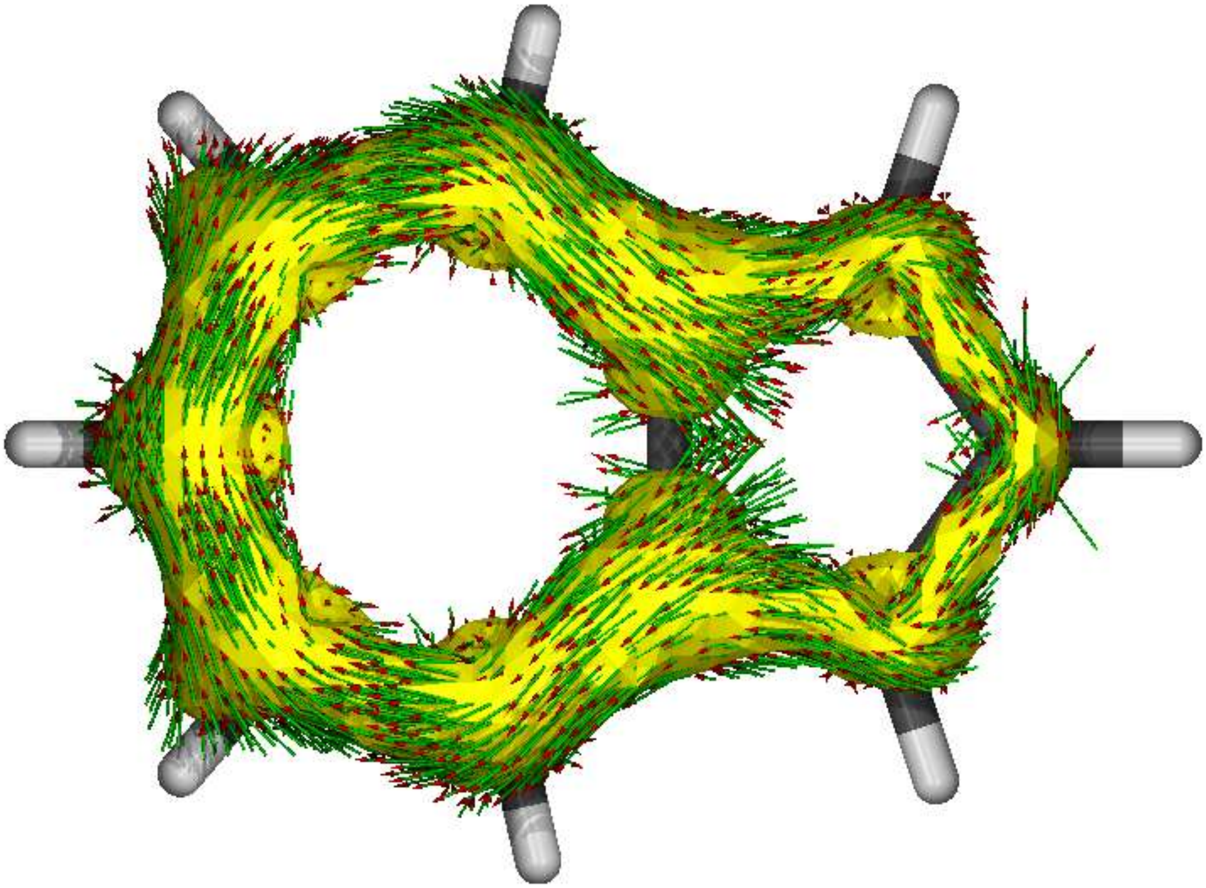

Figure S37: Qu_1 π-ACID plot of azulene, calculated at B3LYP/6-311+G** level of theory.

S15 Full resolution MICD plots

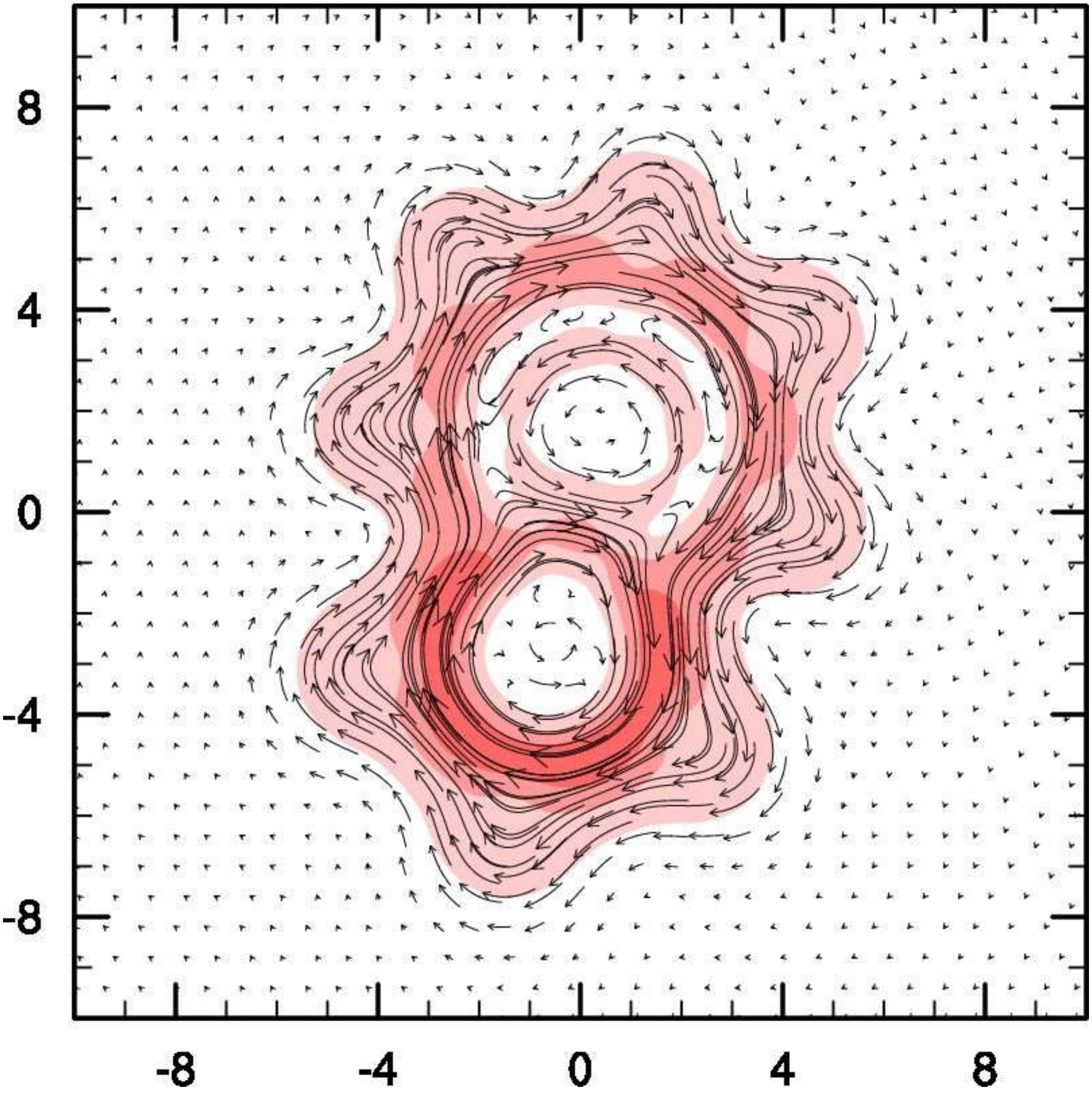

Figure S38: S_0 MICD plot of azulene, constructed 1 a.u. above the molecular plane, calculated at CASSCF(10,10)/TZVP level of theory.

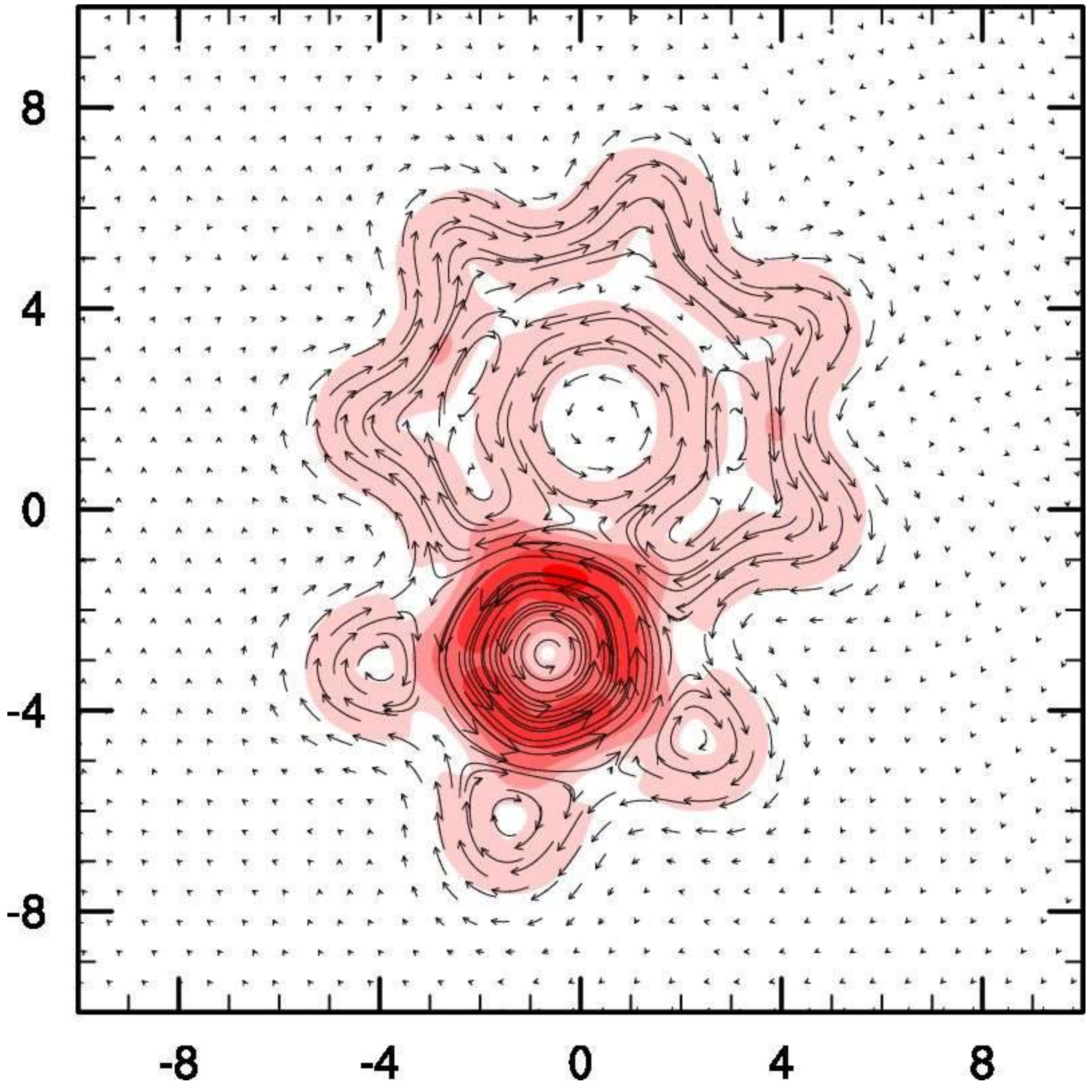

Figure S39: S_1 MICD plot of azulene, constructed 1 a.u. above the molecular plane, calculated at CASSCF(10,10)/TZVP level of theory.

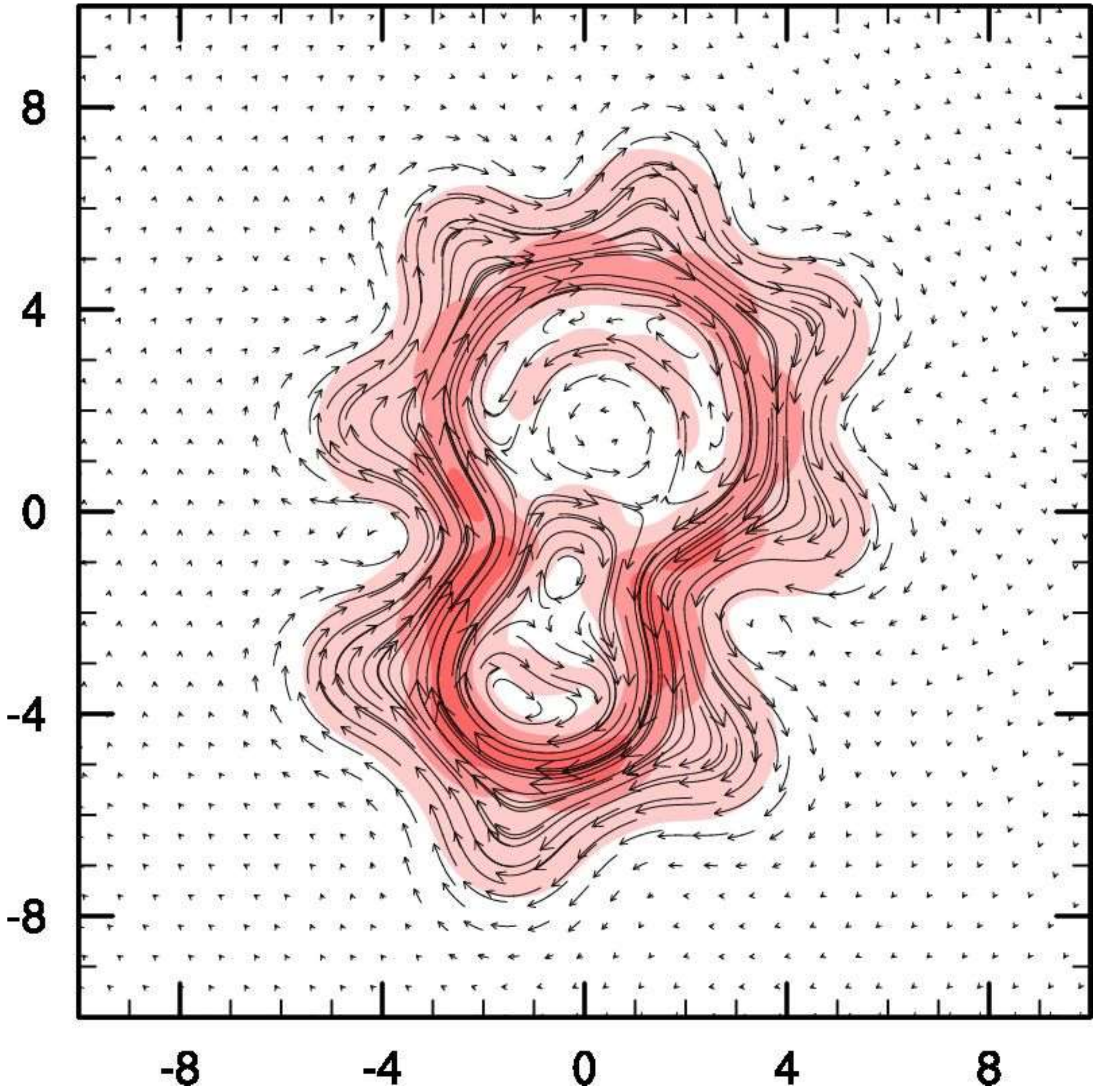

Figure S40: S_2 MICD plot of azulene, constructed 1 a.u. above the molecular plane, calculated at CASSCF(10,10)/TZVP level of theory.

S16 Coordinates in .XYZ format

Coordinates are contained in a separate supplementary file.

S17 References

1. Dewar, M. J. S.; Dougherty, R. C., PMO Treatment of Conjugated Systems. In *The PMO Theory of Organic Chemistry*, Dewar, M. J. S.; Dougherty, R. C., Eds. Springer US: Boston, MA, 1975; pp 73-130.

2. Baird, N. C., Quantum organic photochemistry. II. Resonance and aromaticity in the lowest 3.pi..pi.* state of cyclic hydrocarbons. *Journal of the American Chemical Society* **1972,** *94* (14), 4941-4948.

3. Mandado, M.; Graña, A. M.; Pérez-Juste, I., Aromaticity in spin-polarized systems: Can rings be simultaneously alpha aromatic and beta antiaromatic? *The Journal of Chemical Physics* **2008,** *129* (16), 164114.

4. Soncini, A.; Fowler, P. W., Ring-current aromaticity in open-shell systems. *Chemical Physics Letters* **2008,** *450* (4), 431-436.

5. Solà, M., Aromaticity rules. *Nature Chemistry* **2022,** *14* (6), 585-590.

6. Neese, F.; Wennmohs, F.; Becker, U.; Riplinger, C., The ORCA quantum chemistry program package. *The Journal of Chemical Physics* **2020,** *152* (22), 224108.

7. M. J. Frisch, G. W. T., H. B. Schlegel, G. E. Scuseria, M. A. Robb, J. R. Cheeseman, G. Scalmani, V. Barone, G. A. Petersson, H. Nakatsuji, X. Li, M. Caricato, A. Marenich, J. Bloino, B. G. Janesko, R. Gomperts, B. Mennucci, H. P. Hratchian, J. V. Ortiz, A. F. Izmaylov, J. L. Sonnenberg, D. Williams-Young, F. Ding, F. Lipparini, F. Egidi, J. Goings, B. Peng, A. Petrone, T. Henderson, D. Ranasinghe, V. G. Zakrzewski, J. Gao, N. Rega, G. Zheng, W. Liang, M. Hada, M. Ehara, K. Toyota, R. Fukuda, J. Hasegawa, M. Ishida, T. Nakajima, Y. Honda, O. Kitao, H. Nakai, T. Vreven, K. Throssell, J. A. Montgomery, Jr., J. E. Peralta, F. Ogliaro, M. Bearpark, J. J. Heyd, E. Brothers, K. N. Kudin, V. N. Staroverov, T. Keith, R. Kobayashi, J. Normand, K. Raghavachari, A. Rendell, J. C. Burant, S. S. Iyengar, J. Tomasi, M. Cossi, J. M. Millam, M. Klene, C. Adamo, R. Cammi, J. W. Ochterski, R. L. Martin, K. Morokuma, O. Farkas, J. B. Foresman, and D. J. Fox *Gaussian 09, Revision A.02*, Wallingford, CT, 2016.

8. Aidas, K.; Angeli, C.; Bak, K. L.; Bakken, V.; Bast, R.; Boman, L.; Christiansen, O.; Cimiraglia, R.; Coriani, S.; Dahle, P.; Dalskov, E. K.; Ekström, U.; Enevoldsen, T.; Eriksen, J. J.; Ettenhuber, P.; Fernández, B.; Ferrighi, L.; Fliegl, H.; Frediani, L.; Hald, K.; Halkier, A.; Hättig, C.; Heiberg, H.; Helgaker, T.; Hennum, A. C.; Hettema, H.; Hjertenæs, E.; Høst, S.; Høyvik, I.-M.; Iozzi, M. F.; Jansík, B.; Jensen, H. J. A.; Jonsson, D.; Jørgensen, P.; Kauczor, J.; Kirpekar, S.; Kjærgaard, T.; Klopper, W.; Knecht, S.; Kobayashi, R.; Koch, H.; Kongsted, J.; Krapp, A.; Kristensen, K.; Ligabue, A.; Lutnæs, O. B.; Melo, J. I.; Mikkelsen, K. V.; Myhre, R. H.; Neiss, C.; Nielsen, C. B.; Norman, P.; Olsen, J.; Olsen, J. M. H.; Osted, A.; Packer, M. J.; Pawlowski, F.; Pedersen, T. B.; Provasi, P. F.; Reine, S.; Rinkevicius, Z.; Ruden, T. A.; Ruud, K.; Rybkin, V. V.; Sałek, P.; Samson, C. C. M.; de Merás, A. S.; Saue, T.; Sauer, S. P. A.; Schimmelpfennig, B.; Sneskov, K.; Steindal, A. H.; Sylvester-Hvid, K. O.; Taylor, P. R.; Teale, A. M.; Tellgren, E. I.; Tew, D. P.; Thorvaldsen, A. J.; Thøgersen, L.; Vahtras, O.; Watson, M. A.; Wilson, D. J. D.; Ziolkowski, M.; Ågren, H., The Dalton quantum chemistry program system. *WIREs Computational Molecular Science* **2014,** *4* (3), 269-284.

9. Pracht, P.; Bohle, F.; Grimme, S., Automated exploration of the low-energy chemical space with fast quantum chemical methods. *Physical Chemistry Chemical Physics* **2020,** *22* (14), 7169-7192.

10. Szczepanik, D. W.; Andrzejak, M.; Dominikowska, J.; Pawełek, B.; Krygowski, T. M.; Szatylowicz, H.; Solà, M., The electron density of delocalized bonds (EDDB) applied for quantifying aromaticity. *Physical Chemistry Chemical Physics* **2017,** *19* (42), 28970-28981.

11. E. D. Glendening, J., K. Badenhoop, A. E. Reed, J. E. Carpenter, J. A. Bohmann, C. M. Morales, P. Karafiloglou, C. R. Landis, F. Weinhold *NBO 7.0*, Theoretical Chemistry Institute, University of Wisconsin, Madison 2018.

12. Keith, T. A. *AIMAll v 19.10.12*, TK Gristmill Software: Overland Park KS, USA 2014.

13. Matito, E.; Solà, M.; Salvador, P.; Duran, M., Electron sharing indexes at the correlated level. Application to aromaticity calculations. *Faraday Discussions* **2007,** *135* (0), 325-345.

14. Matito, E. *ESI-3D: Electron sharing indexes program for 3D molecular space partitioning*, IQCC (Girona, Catalonia) and DIPC (Donostia, Euskadi), Spain, 2015.

15. Weigend, F.; Ahlrichs, R., Balanced basis sets of split valence, triple zeta valence and quadruple zeta valence quality for H to Rn: Design and assessment of accuracy. *Physical Chemistry Chemical Physics* **2005,** *7* (18), 3297-3305.

16. Grimme, S.; Antony, J.; Ehrlich, S.; Krieg, H., A consistent and accurate ab initio parametrization of density functional dispersion correction (DFT-D) for the 94 elements H-Pu. *The Journal of Chemical Physics* **2010,** *132* (15), 154104.

17. Grimme, S.; Ehrlich, S.; Goerigk, L., Effect of the damping function in dispersion corrected density functional theory. *Journal of Computational Chemistry* **2011,** *32* (7), 1456-1465.

18. Izsák, R.; Neese, F., An overlap fitted chain of spheres exchange method. *The Journal of Chemical Physics* **2011,** *135* (14), 144105.

19. Bannwarth, C.; Ehlert, S.; Grimme, S., GFN2-xTB—An Accurate and Broadly Parametrized Self-Consistent Tight-Binding Quantum Chemical Method with Multipole Electrostatics and Density-Dependent Dispersion Contributions. *Journal of Chemical Theory and Computation* **2019,** *15* (3), 1652-1671.

20. Yanai, T.; Tew, D. P.; Handy, N. C., A new hybrid exchange–correlation functional using the Coulomb-attenuating method (CAM-B3LYP). *Chemical Physics Letters* **2004,** *393* (1), 51-57.

21. Li, Z.; Liu, W., First-order nonadiabatic coupling matrix elements between excited states: A Lagrangian formulation at the CIS, RPA, TD-HF, and TD-DFT levels. *The Journal of Chemical Physics* **2014,** *141* (1), 014110.

22. Fatehi, S.; Alguire, E.; Shao, Y.; Subotnik, J. E., Analytic derivative couplings between configuration-interaction-singles states with built-in electron-translation factors for translational invariance. *The Journal of Chemical Physics* **2011,** *135* (23), 234105.

23. Guo, Y.; Sivalingam, K.; Valeev, E. F.; Neese, F., SparseMaps—A systematic infrastructure for reduced-scaling electronic structure methods. III. Linear-scaling multireference domain-based pair natural orbital N-electron valence perturbation theory. *The Journal of Chemical Physics* **2016,** *144* (9), 094111.

24. Guo, Y.; Riplinger, C.; Liakos, D. G.; Becker, U.; Saitow, M.; Neese, F., Linear scaling perturbative triples correction approximations for open-shell domain-based local pair natural orbital coupled cluster singles and doubles theory [DLPNO-CCSD(T0/T)]. *The Journal of Chemical Physics* **2020,** *152* (2), 024116.

25. Liakos, D. G.; Neese, F., Is It Possible To Obtain Coupled Cluster Quality Energies at near Density Functional Theory Cost? Domain-Based Local Pair Natural Orbital Coupled Cluster vs Modern Density Functional Theory. *Journal of Chemical Theory and Computation* **2015,** *11* (9), 4054-4063.

26. Fliegl, H.; Taubert, S.; Lehtonen, O.; Sundholm, D., The gauge including magnetically induced current method. *Physical Chemistry Chemical Physics* **2011,** *13* (46), 20500-20518.

27. Bast, R.; Jusélius, J.; Saue, T., 4-Component relativistic calculation of the magnetically induced current density in the group 15 heteroaromatic compounds. *Chemical Physics* **2009,** *356* (1), 187-194.

28. Pathak, S.; Bast, R.; Ruud, K., Multiconfigurational Self-Consistent Field Calculations of the Magnetically Induced Current Density Using Gauge-Including Atomic Orbitals. *Journal of Chemical Theory and Computation* **2013,** *9* (5), 2189-2198.

29. Jusélius, J.; Sundholm, D.; Gauss, J., Calculation of current densities using gauge-including atomic orbitals. *The Journal of Chemical Physics* **2004,** *121* (9), 3952-3963.

30. Scott, L. T.; Brunsvold, W. R.; Kirms, M. A.; Erden, I., Homoazulene. *Journal of the American Chemical Society* **1981,** *103* (17), 5216-5220.

31. Balaban, A. T.; Klein, D. J., Claromatic Carbon Nanostructures. *The Journal of Physical Chemistry C* **2009,** *113* (44), 19123-19133.

32. El Bakouri, O.; Szczepanik, D. W.; Jorner, K.; Ayub, R.; Bultinck, P.; Solà, M.; Ottosson, H., Three-Dimensional Fully π-Conjugated Macrocycles: When 3D-Aromatic and When 2D-Aromatic-in-3D? *Journal of the American Chemical Society* **2022,** *144* (19), 8560-8575.
33. Clar, E., The Aromatic Sextet. In *Mobile Source Emissions Including Policyclic Organic Species*, Rondia, D.; Cooke, M.; Haroz, R. K., Eds. Springer Netherlands: Dordrecht, 1983; pp 49-58.
